# The intrinsically disordered region of GCE protein adopts a more fixed structure by interacting with the LBD of the nuclear receptor FTZ-F1

**DOI:** 10.1186/s12964-020-00662-2

**Published:** 2020-11-05

**Authors:** Marta Kolonko, Dominika Bystranowska, Michał Taube, Maciej Kozak, Mark Bostock, Grzegorz Popowicz, Andrzej Ożyhar, Beata Greb-Markiewicz

**Affiliations:** 1 Department of Biochemistry, Molecular Biology and Biotechnology, Faculty of Chemistry, |Wroclaw University of Science and Technology|, Wybrzeze Wyspianskiego 27, 50-370 Wroclaw, Poland; 2 Department of Macromolecular Physics, Faculty of Physics, Adam Mickiewicz University, Uniwersytetu Poznanskiego 2, 61-614 Poznan, Poland; 3 National Synchrotron Radiation Centre SOLARIS, Jagiellonian University, Czerwone Maki 98, 30-392 Krakow, Poland; 4 Biomolecular NMR and Center for Integrated Protein Science Munich at Department Chemie, Technical University of Munich, Lichtenbergstraße 4, 85748 Garching, Germany; 5 Institute of Structural Biology, Helmholtz Zentrum München, Ingolstädter Landstraße 1, 85764 Oberschleißheim, Germany

**Keywords:** Germ cell-expressed protein, Intrinsically disordered proteins, bHLH-PAS transcription factor, C-terminus, Protein-protein interactions, FTZ-F1

## Abstract

The *Drosophila melanogaster* Germ cell-expressed protein (GCE) is a paralog of the juvenile hormone (JH) receptor - Methoprene tolerant protein (MET). Both proteins mediate JH function, preventing precocious differentiation during *D. melanogaster* development. Despite that GCE and MET are often referred to as equivalent JH receptors, their functions are not fully redundant and show tissue specificity. Both proteins belong to the family of bHLH-PAS transcription factors. The similarity of their primary structure is limited to defined bHLH and PAS domains, while their long C-terminal fragments (GCEC, METC) show significant differences and are expected to determine differences in GCE and MET protein activities. In this paper we present the structural characterization of GCEC as a coil-like intrinsically disordered protein (IDP) with highly elongated and asymmetric conformation. In comparison to previously characterized METC, GCEC is less compacted, contains more molecular recognition elements (MoREs) and exhibits a higher propensity for induced folding. The NMR shifts perturbation experiment and pull-down assay clearly demonstrated that the GCEC fragment is sufficient to form an interaction interface with the ligand binding domain (LBD) of the nuclear receptor Fushi Tarazu factor-1 (FTZ-F1). Significantly, these interactions can force GCEC to adopt more fixed structure that can modulate the activity, structure and functions of the full-length receptor. The discussed relation of protein functionality with the structural data of inherently disordered GCEC fragment is a novel look at this protein and contributes to a better understanding of the molecular basis of the functions of the C-terminal fragments of the bHLH-PAS family.

Video abstract.

Video abstract.

## Background

*Drosophila melanogaster* has become an important model organism in research aimed to understand the molecular basis of organism development, since the fundamental mechanisms and pathways controlling development have been preserved during evolution [1].

Insect growth and development are controlled by the cross-talk between only two hormones: 20-hydroxyecdysone (20E) and juvenile hormone (JH) [2]. While a high JH titer maintains cell divisions without differentiation (morphostasis) [3], the decrease of JH concentration in the hemolymph of the last larval instar stage allows 20E dependent transition to the pupa, and finally metamorphosis [3]. Interestingly, JH participates not only in the development of insects, but it also regulates diverse biological functions during the adult life of an insect, such as: female and male reproduction, pheromone production, migration and diapause [4, 5].

The *Methoprene tolerant* protein (MET), as the JH receptor, mediates the function of JH in preventing the precocious development of *D. melanogaster* during metamorphosis [6]. The deletion of the *met* gene is lethal to most species of insects. However, in *D. melanogaster* there exists a MET paralog - the Germ cell-expressed protein (GCE). As demonstrated, GCE exhibits a high affinity for JH and the ability to take over the MET function in *met* null mutants, ensuring their survival [7]. However, it was shown that MET and GCE functions are not fully redundant and present tissue specificity [8]. The functional unevenness between GCE and MET as transcription factors seems to be exceptionally interesting. These proteins not only exhibit differentiated stage and tissue specific expression, but also different functions during organism development and adulthood. It was shown that the *met* null-mutants of *D. melanogaster* are viable at the pupae stage due to the presence of GCE. However, GCE is not able to adopt MET functions in the eyes or genitals [7]. In contrast, GCE is essential for the proper functioning of the digestive system and is indispensable for the induction of the E75A nuclear receptor expression, which is extremely important during larval development and metamorphosis [5]. Interestingly, only MET overexpression is lethal [9]. GCE and MET also differ in the distribution of the nuclear localization and nuclear export signals (NLSs, NESs, respectively) within protein.. Interestingly, the final localization of GCE seems to be regulated in a much more complex manner than MET [10, 11]. As a result, the differentiated subcellular distribution of MET and GCE during *D. melanogaster* development could be one of the factors responsible for their partially different functions.

**Fig. 1:**
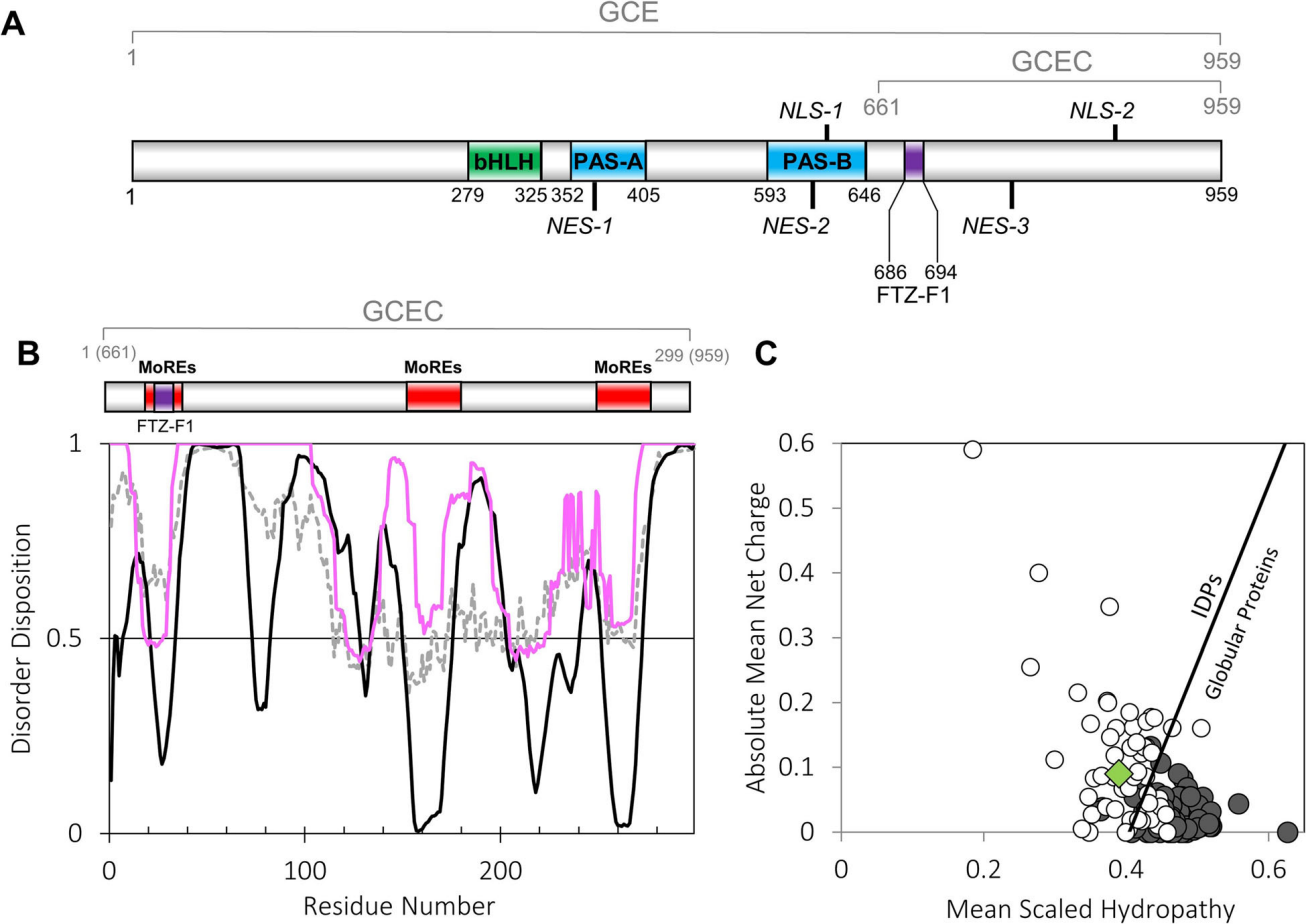
In silico analysis of the GCEC protein sequence. **a** The domain structure of GCE [12]. Green indicates the bHLH domain, whereas blue represents the PAS domains. Violet indicates the interaction site for the FTZ-F1 factor [5]. NLSs and NESs are marked [11]. **b** Predictions of the occurrence of IDRs based on the GCEC amino acid sequence. The top panel represents the localization of the predicted MoREs in GCEC sequence (red color). Violet indicates the interaction site for the FTZ-F1 factor. The bottom panel presents PONDR-VLXT [13] (solid black line), IUPred [14] (dashed black line) and GeneSilico MetaDisorder [15] (solid pink line) prediction results. A score of over 0.5 indicates a high probability of disorder. **c** The Uversky charge-hydropathy plot comparing the mean net charge and the mean hydropathy for disordered (open circles) and ordered proteins (grey circles) [16–18]. The boundary between ordered and disordered proteins is marked. The green diamond corresponds to GCEC.

GCE and MET have been assigned to the family of basic helix-loop-helix/Per-Arnt-Sim (bHLH-PAS) transcription factors (see Fig. 1a), which are responsible for the regulation of important developmental and physiological processes in eukaryotes [12]. bHLH-PAS proteins present a relatively well-conserved domain structure [12]. While the bHLH domain is responsible for DNA binding [19], the PAS-A domain mediates protein-protein interactions and ensures the specificity of target gene activation [12]. The PAS-B domain is responsible for ligand binding and often functions as a signal sensor [12, 20]. The importance of bHLH-PAS proteins for mammalian development and physiology has been carefully presented previously [21].

The similarity between the primary structures of GCE and MET is limited to the defined bHLH and PAS domains, while their long C-terminal fragments (GCEC, METC, respectively) are highly variable. It was documented that the C-termini of bHLH-PAS proteins comprise transcription activation/repression domains (TAD/RPD) [22, 23]. TAD/RPD are responsible for the specific modulation of bHLH-PAS transcription factors and their partners action [12].

As demonstrated, GCE and MET are able to interact with the transcription factor *Fushi Tarazu* factor-1 (FTZ-F1) [24]. *Drosophila* FTZ-F1 plays a critical role in the development of the segmented body plan in the embryo [25] and allows the crosstalk of the 20E and JH signaling pathways. In the absence of JH, FTZ-F1 binds to the 20E receptor composed of two proteins: the Ultraspiracle (Usp) and Ecdysone receptor (Ecr), and contributes to the induction of metamorphosis. Binding to GCE or MET impacts FTZ-F1 activity and allows the expression of specific JH-dependent genes [26]. The interactions involving FTZ-F1 usually depend on canonical charge clamp residues forming hydrogen bonds with a partner protein [24]. The charge clamp, referred to as activation function 2 (AF2), is formed by the FTZ-F1 ligand binding domain (LBD) helixes. However, the interactions between FTZ-F1 and GCE/MET are based on completely different, hydrophobic contact with AF2. For interactions, GCE and MET utilize the novel NR-box (LIXXL motif) present in the C-terminal fragments of both proteins. As shown in [5], the presence of mutations in the area of the GCE/MET LIXXL motif results in a reduced binding ability to FTZ-F1, which confirms the NR-box as the crucial site of interaction. Interestingly, it was shown that the FTZ-F1 – GCE complex is also formed efficiently in the absence of hormone, and that it enables the specific genes expression activation [5]. In contrast, FTZ-F1 – MET complex formation without hormone is inefficient [5].

As mentioned before, it was shown that the C-termini of bHLH-PAS proteins containing TAD/RPD can actively modulate the specificity of these transcription factors function [12]. Taking this into consideration, we hypothesize that the discussed-above structural differences between MET and GCE, especially the differences comprising their long C-termini, could determine the various properties of these two proteins. In this paper we present the structural characterization of GCEC as an intrinsically disordered protein (IDP). It is worth noting that the GCEC region is not fully disordered, since the presence of short fragments adopting more ordered structures was confirmed with denaturation and small-angle X-ray scattering (SAXS) experiments. More ordered fragments, referred to as molecular recognition elements (MoREs), seem to be critical during the protein-protein recognition process, like for the interactions of GCEC with LBD FTZ-F1. Finally, we refer all the GCEC structural data to the previously presented METC characteristic [27]. Importantly, GCEC is defined as less compacted and shows a higher propensity to folding in comparison to METC. The defined structural differences can clearly differentiate the specific functions, subcellular distribution and activity of GCE and MET.

Additionally, we performed NMR spectroscopy and pull-down experiments to analyze the interactions between the LBD of FTZ-F1 and GCEC. All the presented results were consistent and indicated the intrinsically disordered GCEC (or GCE^PEP^ representing the novel GCE NR-box) as sufficient to form an interaction interface with the LBD of FTZ-F1 in vitro*.* Significantly, these interactions can force GCEC to adopt a more fixed structure. We suggest that the GCEC could be sufficient to modulate the FTZ-F1 nuclear receptor activity in a FTZ-F1 LBD dependent manner. We assume that the discussed relation of protein functionality with the structural data of an inherently disordered GCEC fragment is a novel look at this protein, and in consequence at the differences between GCE and its paralog MET.

## Materials and methods

### In silico analysis

The IUPred server (http://iupred.enzim.hu) [14] was used for GCEC intrinsic disorder (ID) predictions. The Uversky plot, PONDR-VLXT [13] and PONDR-VLS2 [28] calculations were made using PONDR (http://www.pondr.com) [13]. Additional analysis was performed using the DISOPRED2 server (http://bioinf.cs.ucl.ac.uk/psipred/) [29] and FoldIndex server (https://omictools.com/foldindex-tool/) [30]. The GeneSilico MetaDisorder server (iimcb.genesilico.pl/metadisorder) [15] was used for averaging of the results. All analyses were performed using default settings.

### Chemicals for GCEC and FTZ-F1 purification

All buffers were prepared using Milli-Q® water and titrated to the final pH at room temperature. The lysis Buffer was 20 mM Tris-HCl and 150 mM NaCl (pH 7.5). Buffer A was PBS (137 mM NaCl, 2.7 mM KCl, 10 mM Na_2_HPO_4_, 1.8 mM KH_2_PO_4_), 0.2% Tween20 and 5 mM β-mercaptoetanol (pH 7.4). Buffer B was 6 M GdmCl_2_ in 20 mM MES and 5 mM β-mercaptoetanol (pH 6.0). Buffer C was 20 mM Tris-HCl, 7.5% glycerol and 5 mM DTT (pH 8.0). Buffer D was 20 mM Tris-HCl, 150 mM NaCl and 5 mM DTT (pH 8.0). Buffer E was the same as buffer D, enriched by 350 mM imidazole. Buffer F was PBS and 2 mM DTT (pH 7.4).

### GCEC and FTZ-F1 expression vector preparation and peptide synthesis

The cDNA encoding full length *D. melanogaster* GCE protein was kindly received from Prof. Thomas G. Wilson (Ohio State University). The cDNA encoding LDB FTZ-F1 (786–1027) was synthesized (Gene Art Thermo Fisher Scientific). Both cDNAs were used as a template during the polymerase chain reaction (PCR). The *E. coli* DH5α strain was used as the host strain during vector preparation. A fragment of the cDNA corresponding to the C-terminus of the GCE (661–959, Fig. S1) was amplified using two primers: forward primer 5′ aaa acc atg gcc ATC AAC ACA CAG A 3′ and reverse primer 5′ aaa agc ggc cgc CTA GTC CTG G 3′. The primers used for LBD FTZ-F1 cDNA amplification were: forward primer 5′ aaa aaa cat atg ATG CTG GAA GAT 3′ and reverse primer 5′ aaa aaa gcg gcg CTA TCC CTT GCG CTT 3′. The primers introduced restriction sites for specific endonucleases (underlined in primer sequences), respectively NcoI and NotI for GCEC, and NdeI and NcoI for LBD FTZ-F1. The upper-case letters in the primer sequence represent the sequence present in the GCEC or LBD FTZ-F1, respectively. The purified PCR products were cloned into pET-M11 (GCEC) or pET-15b (LBD FTZ-F1) vectors, which were digested with the appropriate restriction enzymes. Both fragments were inserted in a frame with the hexahistidine tag (6 × His tag). The final constructs: pET-M11/GCEC and pET-15b/LBD FTZ-F1 sequences were confirmed by DNA sequencing.

The 9-residue GCEC peptide (GCE^PEP^, LRLIQNLQK) was synthesized (PSL GmbH, Heidelberg). The product purity determined by NMR was > 98%.

### Expression and purification of GCEC

The BL21(DE3) *E. coli* strain was used for GCEC expression. Bacteria were transformed with 2 ng of pET-M11/GCEC plasmid and plated on Lysogeny Broth (LB) agar containing 30 μg/ml kanamycin. After overnight incubation at 37 °C, a single colony was used to inoculate 20 ml of LB medium containing 30 μg/ml kanamycin. The culture was incubated overnight at 37 °C in a rotary shaker operated at 182 rpm. 15 ml of starting culture was used to inoculate 500 ml of ZYM-5052 auto-inducing medium or N-5052 auto-inducing minimal medium for ^15^N labeling [31], both supplemented with 100 μg/ml kanamycin. The incubation was conducted at 37 °C until the optical density (OD_600_) reached 2.0. The incubation was continued for 15 h in a temperature reduced to 20 °C. The culture was harvested by centrifugation at 4000×g (20 min, 4 °C), resuspended in 10 ml of lysis buffer supplemented with 0.2 mg/ml phenylmethylsulfonyl fluoride (PMSF) and frozen at − 80 °C. The frozen cells were thawed and supplemented with PMSF in a final concentration of 0.2 mg/ml, β-mercaptoethanol (5 mM), DNase I (20 μg/ml) and RNase A (20 μg/ml). The cell extract was sonicated for 15 min and centrifuged at 20000×g for 1 h at 4 °C. The 6xHis-GCEC was present in the insoluble fraction.

The obtained pellet was washed by resuspending in buffer A and centrifugated for 15 min. This step was repeated three times. Finally, the washed pellet was resuspended in 1 ml of buffer B and incubated at 37 °C with shaking at 182 rpm for 12 h. The obtained suspension was than centrifugated at 20000×g for 1 h at 4 °C. Denatured proteins, including GCEC, were present in the soluble fraction. Finally, GCEC was refolded by dilution. 1 ml of the denatured proteins was added, drop by drop, to 200 ml of buffer C, agitated continuously and incubated at 4 °C for 16 h. Since the expressed recombinant protein had a 6 × His tag, immobilized metal affinity chromatography (IMAC) was used to concentrate the GCEC. 1 ml of Ni^2+^-NTA His-bind resin (Novagen), pre-equilibrated with buffer D, was added to the refolded solution and agitated for 1 h at 4 °C. The resin was collected by loading it on a reusable column (20 ml, Clontech) and then washed with 20 ml of buffer D. The 6xHis-GCEC protein was eluted with 10 ml of buffer E. 1 ml fractions were collected. Selected fractions presenting the highest absorbance (A_280_) were centrifuged at 18000×g for 5 min and loaded on the Superdex200 10/300GL column (Amersham Pharmacia Biotech) equilibrated with Buffer F, connected to an ÄKTAexplorer (Amersham Biosciences). The system was operated at 0.5 ml/min at room temperature and the absorbances at 220 and 280 nm were monitored (Fig. S2A). Samples containing the purified GCEC protein were collected and used for further analysis.

### Expression and purification of FTZ-F1

The BL21(DE3) *E. coli* strain was used for FTZ-F1 expression. Bacteria were transformed with 2 ng of the pET-15b/LBD FTZ-F1 plasmid. The further expression procedure was analogical to the procedure described for the GCEC. The 6xHis-LBD FTZ-F1 was present in the soluble fraction. The supernatant obtained from 1 l culture was passed twice over 3 ml of Ni^2+^-NTA His-bind resin (Novagen), pre-equilibrated with buffer D. After binding, the resin was washed with 20 ml of buffer D. The 6xHis-LBD FTZ-F1 protein was eluted with 10 ml of buffer E. Protease (Thrombin) was used to remove the 6xHis tag. 1 mg of Thrombin was added to the eluted protein, mixed gently and incubated overnight at 4 °C. The buffer was exchanged to buffer D and a second step of IMAC was performed to remove the 6xHis-tag and Thrombin. Fractions containing LBD FTZ-F1 were concentrated, centrifuged at 18000×g for 5 min and loaded on the Superdex75 10/300GL column (Amersham Pharmacia Biotech) pre-equilibrated with buffer F (Fig. S3A). Purified FTZ-F1 electrophoretic mobility is appropriate for 26.5 kDa globular protein (Fig. S3B). The obtained preparation was stable and then used for further analyzes.

### Sodium dodecyl sulfate polyacrylamide gel electrophoresis (SDS-PAGE)

Samples collected during GCEC and LBD FTZ-F1 expression and purification were analyzed using SDS-PAGE (12% polyacrylamide gels developed in a Tris/glycine system [32]). The Precision Plus ProteinTM Standards Weight Marker (Bio-rad) was used as a molecular mass (MM) protein standard. The gels were stained with SimplyBlue™ SafeStain (Invitrogen).

### Determination of protein concentration

The purified protein concentrations were measured spectrophotometrically at 280 nm. GCEC (0.25) and LBD FTZ-F1 (0.84) absorption coefficients were calculated based on the amino acid (aa) sequence using the ProtParam tool [33], available at http://us.expasy.org/tools/protparam.html.

### Protein identity confirmation

The identity of the obtained GCEC samples was confirmed using Electrospray ionization (ESI) mass spectrometry, as described previously [27]. Additionally, we performed GCEC protein sequencing (Sanger sequencing method [34]).

### Circular dichroism (CD) spectroscopy of GCEC

CD spectra were recorded as described previously [27]. A JASCO J-815 CD-spectropolarimeter with the sample cell temperature control unit (Peltier Type Control System) was used. All scans were performed at 20 °C in 2 mm path-length cuvette 100QS (Hellma) with 20 nm/min speed and a data resolution of 1.0 nm in the spectral range of 190–260 nm. The GCEC concentration was 20 μM. The reference spectrum was recorded in buffer F. Additional measurements were performed after a 1 h of incubation with guanidine hydrochloride (GdmCl) or 2,2,2-trifluoroethanol (TFE). Temperature denaturation spectra were recorded in the temperature range 20–80 °C, at 10 °C intervals. All results with an acceptable high tension (HT under 750 V) were converted to molar residual ellipticity units. For quantitative CD spectrum deconvolution, CDPro spectra software was used (CONTINLL algorithm on the SDP48 base) [35].

### Hydrodynamic analysis of GCEC

Size-exclusion chromatography (SEC) was conducted using the Superdex200 10/300 GL column (Amersham Pharmacia Biotech) connected to the ÄKTAexplorer (Amersham Biosciences) system, which was operated at 0.5 ml/min at room temperature. The UV absorbances at 220 and 280 nm were monitored for protein elution profile determination. The column was equilibrated with buffer F and calibrated with standard proteins: thyroglobulin (669 kDa, 75.1 Å), apoferritin (443 kDa, 64.8 Å), β-amylase (200 kDa, 48.8 Å), alcohol dehydrogenase (150 kDa, 44.0 Å), albumin (66 kDa, 32.9 Å), and carbonic anhydrase (29 kDa, 24.5 Å). Eq 1 was used for the proteins’ Stokes radii (R_S_) calculation [36]. The column void volume (V_0_) determined with blue dextran was 8.54 ± 0.08 ml, and the column total volume (V_T_) was 24 ml. The observed elution volume (V_E_) of each standard protein was used for calculation of the gel-phase distribution coefficients (K_AV_ factors) (Eq. 2 [37]). All determined K_AV_ values were plotted against the calculated R_S_ values. Finally, 0.1 ml of the purified GCEC (1 mg/ml) was loaded on the column. The determined standard curve was used to calculate the GCEC R_S_.

1 $$ \log \left({R}_S\right)=-\left(0.204\pm 0.023\right)+\left(0.357\pm 0.005\right)\bullet \log (MW) $$

2 $$ {K}_{AV}=\frac{V_E-{V}_0}{V_T-{V}_0} $$

Sedimentation velocity (SV) experiments were performed using the Beckman Coulter ProteomeLab XL-I ultracentrifuge (Beckman Coulter Inc.) equipped with an AN-60Ti rotor and cells with 12 mm path-length charcoal-filled two-channel Epon centre pieces. All the experiments were conducted at 20 °C at 50000 rpm, and the absorbance scans were collected at 230 nm. The volume of the samples was 400 μl and the GCEC concentration was 0.07, 0.18 and 0.33 mg/ml in buffer F. The time-corrected scans of the sedimentation process were analyzed using SEDFIT (http://www.analyticalultracentrifugation.com) [38, 39]. The buffer density and dynamic viscosity were calculated using SEDNTERP software (http://sednterp.unh.edu/) [40]. The sedimentation coefficients (S) and the frictional ratios (f/f_0_) were calculated using sedimentation coefficient distribution function [(c(S)]. The maximum-entropy regularization of the c(S) model was set to a confidence level of 0.68. The sedimentation coefficients were corrected to standard conditions (S_20,w_). The plots of the SV data were obtained using GUSSI (version 1.4.2) software [41].

### Small angle X-ray scattering (SAXS)

Small-angle X-ray scattering studies of the GCE C-terminal fragment in solution were performed using the laboratory SAXS/WAXS Xeuss 2.0 system (XENOCS, Sassenage, France) installed on a high brilliance MetalJet D2 microfocus X-ray source (λ = 0.134 nm) with a liquid metal (gallium alloy) target (Excillum AB, Kista, Sweden). 30 μl of the GCEC sample at 0.68 mg/ml, purified freshly by SEC chromatography in buffer F, were injected into a low noise flow cell manually and measurements were performed at 22 °C. Three independent frames (exposition time per frame 600 s) were recorded with the PILATUS 3R 1 M hybrid photon counting detector (Dectris AG, Baden-Daettwil, Switzerland) in order to avoid protein aggregation. All SAXS data were collected over the scattering vector s range from 0.010 to 0.213 Å^− 1^. Data reduction and buffer subtraction were performed using the Foxtrot package [42]. The detailed procedure of data collection and processing was similar to our previous experiments [43, 44]. The radius of gyration (R_g_) value and the pair distance distribution function p(r) calculations were performed with the Primus [45] and GNOM [46] programs from the ATSAS 3.0.1 package [46], respectively. The global conformation of GCEC molecules were also analyzed by an ensemble optimization method (EOM) [47]. First, a pool of 10,000 random conformers based on the protein sequence was generated, and then a genetic algorithm was used to select the GCEC models which exhibit the best fit to the experimental data [48].

### Nuclear magnetic resonance spectroscopy (NMR)

All NMR spectra were collected using the Bruker Avance III (800 MHz) spectrometer equipped with a Superconducting Magnet (Bruker, induction of 18.8 T), pulsed-field gradient system (PFG) Performa I, and cryoprobe for high sensitivity. The protein samples were prepared in buffer F with the addition of 10% D_2_O to provide a lock signal. The samples’ volume was 160 μl and the GCEC concentration was 100 μM. The ^1^H-^15^N spectra were obtained using the HSQC pulse sequence (Heteronuclear Single Quantum Coherence). All measurements were performed in 3 mm NMR tubes (Bruker) at 22 °C. The final spectra were obtained by recording 32 repeats for each of the 256 increments of the t1 time. The relaxation time was 1 s. The total measurement time was 4 h 20 min. The spectral width for the proton dimension was about 2500 Hz, and for the nitrogen channel it was about 11,000 Hz.

Additional spectra of the GCEC and LBD FTZ-F1 were recorded: the labeled GCEC spectrum after protein incubation with an equimolar quantity of unlabeled LBD FTZ-F1, and also the labeled LBD FTZ-F1 spectrum after incubation with the LRLIQNLQK peptide, which corresponds to the binding sequence in the primary structure of the GCEC. The peptide concentration was determined by its solubility limit.

### GCEC and LBD FTZ-F1 vectors for transfection preparation

cDNA fragments corresponding to the GCEC and LBD FTZ-F1 were subcloned into the selected restriction sites of the multiple cloning site (MCS) of the pEYFP-C1 vector for the GCEC (Clontech), and the pECFP-C1 vector for the LBD FTZ-F1 (Clontech). All the primers’ sequences are presented in the supplementary materials (Fig. S4). The primers used for GCEC cDNA amplification introduced the C-terminal FLAG protein sequence (DYKDDDDK). All constructs were verified by DNA sequencing.

### Cell culture and DNA transfection

African green monkey kidney fibroblasts COS-7 (ATCC CRL-1651) were cultured in Dulbecco’s modified Eagle’s medium (DMEM) supplemented with 1% non-essential amino acids (Gibco/Invitrogen), 1 mM sodium pyruvate and 2% glutamine (Gibco/Invitrogen), 10% fetal calf serum (FCS), 100 U/ml penicillin and 100 μg/ml streptomycin. For transfection, the cells were grown at Ø6 cm plates at 37 °C in a 95% air/5% CO2 atmosphere. The cells were transfected with 9 μg of appropriate vectors encoding the GCEC or LBD FTZ-F1 cDNA, or co-transfected with 6 μg of a vector encoding the GCEC and 6 μg of the vector encoding the LBD FTZ-F1. Xfect Transfection Reagent (Takara Bio) was used according to the manufacturer’s instructions. The empty pEYFP-C1 and pECFP-C1 vectors were used as a control.

### FLAG pull-down assay

After 24 h of incubation, all the plates were placed on ice. The medium was removed, and the cells were washed twice with ice cold PBS. After washing, 600 μl of ice-cold lysis buffer (25 mM Tris-HCl, pH 7.4, 150 mM NaCl, 1% NP-40, 1 mM EDTA, 5% glycerol) supplemented with protease and phosphatase inhibitors: PMSF, cOmplete Mini EDTA free Protease Inhibitor Coctail (Roche), Sodium molybdate (Sigma-Aldrich) and Sodium Orthovanadate (Sigma-Aldrich) was added. After 5 min of incubation with periodic mixing, the lysates were transferred to a microcentrifuge tube and centrifugated at 13000 x g for 15 min at 4 °C. The obtained soluble fractions were incubated for 2 h on ice with 20 μl of EZview™ Red ANTI-FLAG M2 Affinity gel (Sigma-Aldrich) pre-equilibrated with TBS buffer (50 mM Tris-HCl, 150 mM NaCl, pH 7.4). After incubation, the gel was washed four times with 500 μl of TBS buffer. Finally, the gel was incubated with 100 μl of elution buffer (TBS buffer supplemented with 100 μg/ml FLAG peptide, Sigma-Aldrich) for 30 min on ice. The eluted proteins were collected for further analysis.

### Western blot analysis

All the samples obtained during the FLAG Pull-down assay were separated by SDS-PAGE using 12% gels and transferred to the Whatman Protran nitrocellulose transfer membrane (Protran BA85, Schleicher & Schuell Pure, Sigma-Aldrich) in the semi-dry system at 10 V for 40 min in Towbin buffer (25 mM Tris, 192 mM glycine, 10% methanol, pH 8.3). The membranes were blocked at room temperature with 2% milk powder (Milchpulver, blotting grade, Roth) in the PBS buffer and incubated for 1 h at room temperature. Next, the membrane was incubated overnight at 4 °C with the specific primary anti-GFP polyclonal antibodies (Sigma-Aldrich) (diluted 1:300 with milk buffer), which cross-react with CFP and YFP. After washing (PBS supplemented with 0.02% Tween, 3 × 10 min), the membrane was incubated for 2 h with secondary goat anti-mouse antibodies coupled to horseradish peroxidase (Vector Laboratories, dilution 1:10000 with milk buffer). Specific signals were detected using the SuperSignal™ West Pico PLUS Substrate Chemiluminescence kit (Thermo Scientific™) according to the manufacturer’s manual. Finally, the membranes were exposed to Kodak BioLight film.

### Fluorescence microscopy

Fluorescence microscopy was performed 24 h after the cells’ transfection in 6-cm diameter Petri dishes in DMEM using an Olympus IX71 microscope with a CFP or YFP filter 24 h after transfection. All the presented images are representative for more than 95% of the observed cells’ population. The empty pEYFP-C1 and pECFP-C1 vectors were used as a control.

## Results

### In silico analyses

It was documented that the C-termini of the bHLH-PAS transcription factors are responsible for the specific modulation of these proteins’ action [12]. Specific chain flexibility, predicted for most of the bHLH-PAS C-termini [21], may be a useful protein feature. To determine to what extent the *D. melanogaster* GCEC structure is disordered, we performed in silico analysis. We used different predictors of protein disorder: PONDR-VLXT [13], PONDR-VLS2 [28], DISOPRED2 [29], FoldIndex [30], IUPred [14] and GeneSilico MetaDisorder [15] to get the full spectrum of possible results. Since all results were comparable, we decided to only show two representative results, and in addition the result of GeneSilico MetaDisorder as a meta-server combining 13 existing methods of prediction (Fig. 1b). The GCEC seems to be mostly disordered along the entire length of the sequence. Short fragments with a tendency to order occur mainly in the area near 30 aa, between 150 and 200 aa, and near 260 aa (predicted with a high probability on the PONDR-VLXT server, Fig. 1b, top panel), and could participate in the protein-protein interactions (PPIs) or act as the molecular recognition elements (MoREs, indicated in red color).

The amino acid composition is one of the factors determining the final conformation adopted by the protein in solution [16–18]. While globular proteins are characterized by a high content of hydrophobic residues and a high hydrophobicity, intrinsically disordered proteins (IDPs) or intrinsically disordered regions (IDRs) are characterized by a high content of charged residues, causing a high net charge. The Uversky diagram [16–18] plots the mean net charge versus the mean hydrophobicity and distinguishes IDPs from ordered proteins (Fig. 1c). Both parameters determined for the GCEC (average hydrophobicity 0.4082 and average charge 0.0769) fit to the values typical for IDPs, which indicates that the GCEC sequence may present the characteristics of IDPs (Fig. 1c).

As the presented results of the in silico analyzes suggested the disordered nature of GCEC, we decided to perform structural characterization of the purified protein in vitro.

### GCEC expression and purification

To perform the GCEC analysis in vitro, we developed and optimized an expression and purification protocol. We tested many vectors, introducing additional tags, which usually improve protein stability and solubility (like TrxA, MBP, SUMO and others), and different bacterial strains. Unfortunately, under all the tested conditions, we were not able to obtain GCEC in a soluble form (data not shown). This may be explained by the toxicity of this protein for bacteria, or by its disordered structure, which results in the formation of inclusion bodies. Consequently, we decided to develop a GCEC purification procedure under denaturing conditions. We focused on the pET-M11 vector, introducing a short polyhistidine tag (6xHis). After protein denaturation with GdmCl, the GCEC was refolded by dilution. The subsequent purification process was simplified, since the inclusion bodies contained mainly recombinant GCEC, with only a small amount of impurities [49]. We used Ni^2+^-NTA resin for the next step of purification. It enabled the refolded GCEC volume to be reduced to 3 ml, a volume equal to the volume of elution, what simultaneously it concentrated the protein. As the final step of purification, we used SEC (Fig. S2A). To verify whether the obtained GCEC sample had the correct molecular mass (MM), we performed ESI mass spectrometry measurements. Two MM values were obtained: 36003 Da, which is compliant with the MM of the construct calculated based on the aa sequence using the ProtParam tool, and also 36,020 Da, which is oversized by 16 Da (in relation to the calculated one). Finally, we performed protein sequencing, which confirmed the GCEC identity and revealed the oxidation of two M residues: M731 (M71 in GCEC) and M909 (M249 in GCEC). The modified form of the GCEC accounted for about 16% of the preparation and appeared with every purification (data not shown).

Purified GCEC appeared as a single band on the 12% SDS-PAGE gel (Fig. S2B). Its electrophoretic mobility was decreased and corresponded to the 42 kDa protein instead of the expected 36 kDa. Such behavior is often observed for IDPs [16, 50]. Their unique amino acid composition has an impact on SDS binding, which results in an unusual mobility in the SDS-PAGE experiments [16, 50]. Existence of purified GCEC in the native, active form is ensured by the ability of GCEC to interact with FTZ-F1 (see below).

### Hydrodynamic analysis of GCEC

**Fig. 2:**
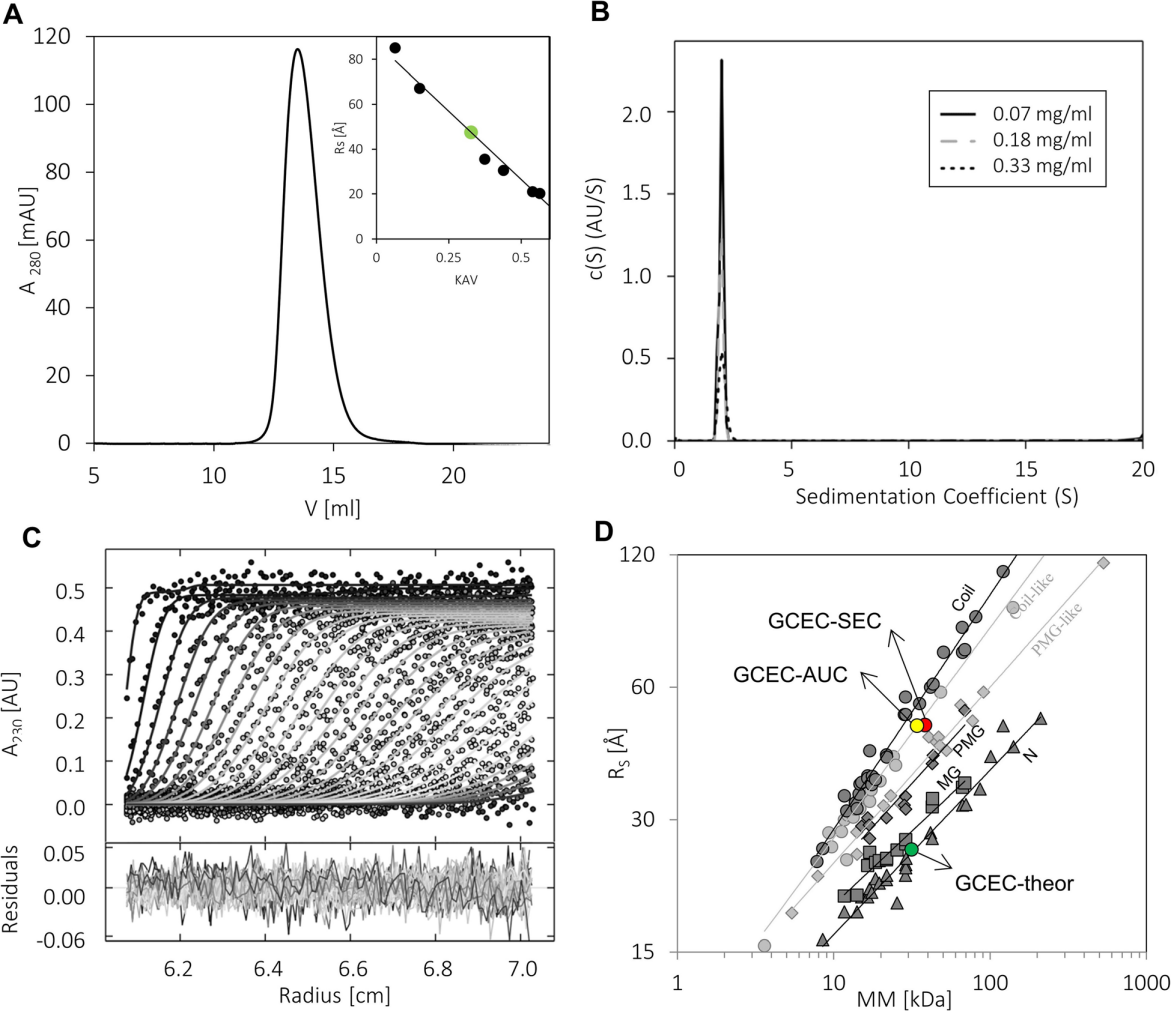
Hydrodynamic properties of GCEC. **a** Analytical SEC of GCEC performed on a Superdex200 10/300 GL column. The graph presents the elution volume of GCEC in a concentration of 1 mg/ml. The inset represents the standard curve determined with standard proteins (black dots). The green circle corresponds to the GCEC. **b** AUC analysis. The graph presents the sedimentation coefficient distributions c(S) for the GCEC at three concentrations: 0.30 mg/ml (black solid line), 0.18 mg/ml (grey dashed line), and 0.07 mg/ml (black dotted line). All data were collected at 230 nm. **c** The representative example of the GCEC sedimentation profile. Selected experimental (circles) and fitted SV profiles (solid lines for GCEC at 0.30 mg/ml) are shown. **d** The relationship between the hydrodynamic radii (R_S_) and the relative MMs determined for four globular proteins states (dark grey) and two IDPs states (light grey) [51, 52]. The globular proteins states are: native proteins (N, tringles), molten globules (MG, squares), pre-molten globules (PMG, diamonds), and 6 M GdmCl-unfolded proteins (coil, circles). The two IDPs states are: coil-like (circles), and PMG-like (diamonds). The theoretical value for GCEC (green dot) and experimental values (SEC – red dot or AUC yellow dot) are shown

One of the easiest ways to identify IDPs is the determination of a protein’s hydrodynamic properties, since IDPs present a significantly overestimated hydrodynamic radius in comparison to globular proteins of the same MM [53]. During analytical size exclusion chromatography (SEC), GCEC was eluted as a single peak with an elution volume corresponding to a R_S_ of 44.7 ± 0.3 Å (Fig. 2a, Table 1). The value was approximately 70% higher than the Rs calculated with the assumption of GCEC globular conformation (26.5 Å, Table 1), and was GCEC concentration independent (data not shown). Therefore, the experimentally determined volume (374.1 Å^3^) of the GCEC was much higher than the theoretical volume (77.9 Å^3^), and the experimentally determined density (0.10 kDa/ Å^− 3^) was much lower than the theoretical density (0.46 kDa/Å^− 3^) (Table 1). This experiment indicated that GCEC has a significantly elongated, poorly packed conformation. However, it was not possible to clearly state if GCEC exists in a monomeric form in solution. The overstated R_S_ value may also be a consequence of protein oligomerization.

**Table 1:** Characterization of GCEC by SEC

MM [kDa]	Rs [Å]		V_**S**_·10^**3**^ [Å^**3**^]		p·10^**− 3**^ [kDa/Å^**− 3**^]	
	theor^**a**^	exp	theor^**b**^	exp^**c**^	theor^**b**^	exp^**c**^
36.0	26.5	44.7	77.9	374.1	0.46	0.10

^a^Calculated from the equation: log(R_S_) = (0.085 ± 0.031) + (0.395 ± 0.016)log(MM) [36]

^b^Calculated using the theoretical R_S_

^c^Calculated using the experimental R_S_

To definitively determine if GCEC can form oligomers, we performed analytical ultracentrifugation (AUC) experiments. We analyzed GCEC samples in three concentrations: 0.07, 0.18 and 0.33 mg/ml. The use of relatively low concentration ranges resulted from the data recording at 230 nm. It was determined by the low absorbance coefficient of the GCEC, in which the aa sequence is characterized by the low content of aromatic aa residues, and in particular no W residues (Abs^280^_0.1%_ = 0.255 ml/(mg∙cm) calculated on the ProtParam server). The very high (above 1.0 AU) absorbance at 230 nm for the samples in higher concentrations would result in huge data errors.

The determined root-mean-square deviation (rmsd = 0.015, Table 2) values were relatively high, which could be the result of the presence of DTT in the buffer. The addition of DTT, which is highly unstable and in reduced form absorbs near 210 nm, may lead to the strong background during analysis in the absorption detection system [54]. GCEC was observed as a single signal at the 2S value (Fig. 2b, Table 2). Importantly, no signal at high S-values, characteristic for oligomers and aggregates, was detected (Fig. 2b). The values of the sedimentation coefficient (S_20,w_) were GCEC concentration independent (Table 2). The experimentally determined R_S_ was approximately 45 Å (Table 2) and was consistent with the SEC result (44.7 ± 3.0 Å). Because of the relatively high rmsd, we decided to perform an additional experiment exploiting the Rayleigh interference detection system. This detection system significantly improves the results of the measurement of samples containing highly absorbing components, such as ATP/GTP and oxidized DTT [54]. We measured the GCEC at two concentrations: 0.31 and 0.82 mg/ml. The main signal corresponded equally to the result obtained using the absorption detection system (S = 2S, Table 3). Importantly, the rmsd value significantly decreased to a value of 0.006 (Table 3), which confirmed a very good fit of results. Again, no signal at high S-values was observed (not shown).

**Table 2:** Characterization of GCEC by sedimentation velocity AUC using an absorption detection system

Concentration [mg/ml]	rmsd	f/f_**0**_	S_**20,w**_ (S)	S (S)	Rs [Å]	App MM [kDa]
0.07	0.01435	2.09	2.118	2.043	46.1	38.3
0.18	0.01517	2.09	2.063	1.990	44.8	36.2
0.33	0.01606	2.03	2.069	1.995	43.8	35.5

**Table 3:** Characterization of GCEC by sedimentation velocity AUC using a Rayleigh interference detection system

Concentration [mg/ml]	rmsd	f/f_**0**_	S_**20,w**_ (S)	S (S)	Rs [Å]	App MM [kDa]
0.31	0.00566	2.01	2.041	1.966	42.6	34.1
0.82	0.00635	2.06	2.009	1.935	44.2	34.8

The frictional ratio f/f_0_ represents the degree of deviation of the molecule from a minimum possible value of 1.0 for a hard, incompressible sphere [55]. Therefore, it allows for protein shape characterization [56]. For globular proteins, f/f_0_ is typically 1.05–1.30 [57]. For IDPs, the f/f_0_ ratio is much higher (1.75–3.0) and increases significantly with the MM [56]. The f/f_0_ calculated for GCEC using AUC data was over 2 (Table 2). This indicates a highly asymmetric and elongated shape, assigning GCEC to coil-like IDPs [56]. The experimentally determined MM is equal to the theoretical molecular weight calculated on the ProtParam server (36.7 kDa vs 36,003.0 Da, Table 2). To conclude, GCEC is a monomeric protein with a highly elongated shape and a high degree of asymmetry.

Analyzing the dependence of the R_S_ on the relative MM, globular proteins can be divided into four states: native proteins (N), molten globules (MGs), pre-molten globules (PMGs) and 6-M GdmCl-unfolded proteins (coil). Two additional IDP states are known: coil-like IDPs) and pre-molten globules-like IDPs (PMG-like) [51, 52]. R_S_ determined for the GCEC with the SEC and AUC experiments place GCEC on the plot relating R_S_ and MM in the area occupied by coil-like IDPs (Fig. 2d). Such a result is consistent with previous in silico and SEC analysis. We performed additional calculations based on equations derived by Tcherkasskaya et al. [57], correlating the MM and the R_S_ for different conformational states of the protein. For GCEC (MM of 36.0 kDa), R_S_ calculated with the assumption of the PMG-like conformation was 36.4 ± 0.4 Å, and of the coil-like it was 51.6 ± 0.7 Å. The experimentally determined R_S_ (44.7 Å SEC and 45 Å AUC) indicates that GCEC conformation corresponds to coil-like IDPs.

Based on hydrodynamic analyzes, we conclude that GCEC exhibits IDPs properties. It has a highly elongated shape, does not oligomerize in solution, and can be assigned to coil-like IDPs.

### Far-UV CD analysis

**Fig. 3:**
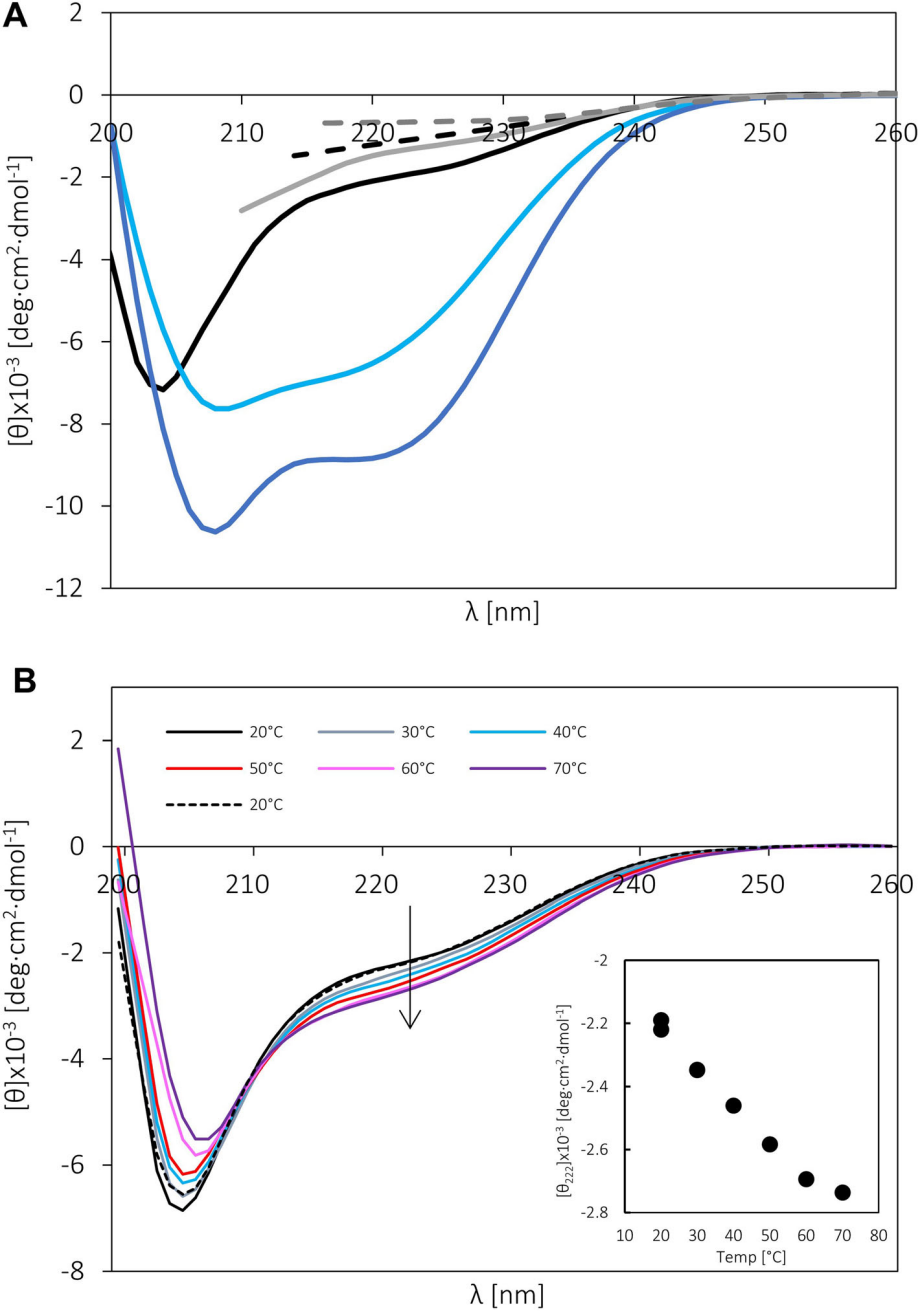
The far-UV CD spectra of GCEC. **a** The CD spectra recorded in buffer F at 20 °C for GCEC: the reference spectrum (black solid line), the spectrum in the presence of 15% TFE (blue solid line) or 30% TFE (dark blue solid line), and the spectrum in the presence of 1 M GdmCl (grey solid line), 2 M GdmCl (black dashed line) or 4 M GdmCl (grey dashed line). **b** The CD spectra of GCEC recorded in buffer F at different temperatures. Inset: dependence of molar residual ellipticity at 222 nm on the temperature. The linear character of the plot indicates no cooperative transition between extreme conformational states

CD spectroscopy is commonly used for the determination of the secondary structure content and folding properties of proteins [58]. The shape of the curve makes it easy to distinguish between α-helical structures (negative peaks at 222 nm and 206 nm) [59], β-strands (negative peaks at 218 nm) [60] and non-regular secondary structures (negative peak at near 200 nm) [61]. The CD spectrum of GCEC (Fig. 3a, Table 4) shows a clear minimum near 200 nm (− 7.2 × 10^− 3^ deg·cm^2^·dmol^− 1^) and a small negative signal near 222 nm (− 1.9 × 10^− 3^ deg·cm^2^·dmol^− 1^). Such a result indicates the disordered character of GCEC and highlights the presence of a residual ordered structure. Deconvolution of the CD spectrum performed with CDPro software (CONTIN/LL algorithm, SPD48 base) confirmed that GCEC is mainly disordered (49.0 ± 5.5%). It also revealed the existence of some ordered structures, mainly β-strands (31.9 ± 6.0%), partially distorted (9.2 ± 2.5%) (Table 4). Moreover, small amounts of totally distorted (5.4 ± 3.7%) α-helixes are estimated (Table 4).

**Table 4:** Characterization of GCEC by CD

Factor	α-helises (%)			β-strands (%)			Turns (%)	U^**a**^ (%)
	R^**a**^	D^**b**^	Σ	R^**a**^	D^**b**^	Σ		
–	0.5 ± 1.6	4.9 ± 2.1	5.4 ± 3.7	22.7 ± 3.5	9.2 ± 2.5	31.9 ± 6.0	13.8 ± 1.5	49.0 ± 5.5
15% TFE	8.7 ± 3.2	8.9 ± 1.5	17.6 ± 4.7	16.9 ± 1.6	9.7 ± 2.1	26.2 ± 3.7	19.4 ± 2.0	36.4 ± 2.0
30% TFE	14.9 ± 6.2	12.8 ± 2.3	27.2 ± 8.5	10.7 ± 3.0	7.7 ± 2.8	18.4 ± 5.8	19.1 ± 3.4	34.3 ± 1.1
1 M GdmCl	0.2 ± 2.5	1.8 ± 3.7	2.0 ± 6.2	14.1 ± 3.4	8.7 ± 3.1	22.8 ± 6.5	12.8 ± 5.4	62.3 ± 4.8
2 M GdmCl	0.0 ± 4.0	2.0 ± 5.2	2.0 ± 9.2	11.5 ± 5.1	6.4 ± 5.7	17.9 ± 10.8	10.0 ± 6.0	68.1 ± 5.2
4 M GdmCl	–	–	–	–	–	–	–	79.8 ± 7.8

^a^ Regular structure

^b^ Distorted structure

^c^ Unstructured

The changes in the CD spectrum observed in the presence of denaturing agents can provide important information regarding protein structure and the degree of protein compaction [62, 63]. To determine the impact of denaturing agent on the GCEC’s secondary structure, we recorded spectra in the presence of 1 M, 2 M and 4 M GdmCl (Fig. 3a). All data, due to the strong absorbance of GdmCl in high concentrations, were collected in a narrow wavelength interval. For this reason quantitative data deconvolution was not performed. The presence of GdmCl resulted in signal blanking at 222 nm (to − 0.6 × 10^− 3^ deg·cm^2^·dmol^− 1^ in the presence of 4 M GdmCl, Fig. 3a). Such an observation clearly confirmed the presence of the residual ordered secondary structure in GCEC in the absence of GdmCl. After incubation with the denaturing agent, the GCEC conformation becomes much more disordered, indicating the loss of the residual ordered secondary structure.

As demonstrated, the temperature and selected chemical reagents (i.e. osmolytes, binding partners, crowding agents, counter ions) can affect the structure of some IDPs [63]. Usually, a more ordered structure can be observed. To determine GCEC conformation changes under certain conditions, the corresponding CD spectra were collected after incubation with TFE or in the function of temperature increase (Fig. 3a and b). First, we studied the influence of 15 and 30% TFE, which is known as ordered secondary structure stabilizer [64]. The presence of TFE significantly affects the shape of the GCEC CD spectrum: the signal around 200 nm decreases, while negative signals around 222 nm and 206 nm, characteristic for ordered secondary structures, appear (Fig. 3a). Data deconvolution revealed a significant increase in the content of α-helical structures (from 5.4 ± 3.7% to 17.6 ± 4.7% and 27.2 ± 8.5% for 15 and 30% TFE respectively, Table 4). Simultaneously, a decrease in the content of β-type structures was observed (Table 4). We suppose that some of the β-structures can be transformed into α-type structures, which is often observed for TFE [65, 66]. However, the decrease in the quantity of β-structures and the increase in the quantity of α-helixes are not proportional and some of the α-helices can be formed from the disordered GCEC fragments. Finally, in the presence of 30% TFE, a significant part of the GCEC (34.3 ± 1.1%) still exhibits a disordered character (Table 4).

Some of coil-like and PMG-like IDPs present a unique temperature response. In contrast to globular proteins, which denature in higher temperatures, such IDPs in the same conditions can adopt a more ordered conformation [63]. This can be explained by the increase of the strength of hydrophobic interactions promoting protein folding [63]. Such behavior can be observed for GCEC. In spectra recorded for GCEC as a function of temperature increase, the signal around 200 nm was gradually reduced and shifted toward higher wavelengths (Fig. 3b). In addition, a characteristic negative maximum around 222 nm appeared, indicating an increase in the content of ordered secondary structures (Fig. 3b). Importantly, these induced structural changes were completely reversible. After cooling the sample to 20 °C, the GCEC spectrum returned to its original shape (Fig. 3b). Since the signal changes observed at 222 nm are linear, there is no cooperative transition between extreme conformational states (Fig. 3b, inset). We analyzed the obtained data with CDPro software, however the observed changes were not big enough to get quantitative deconvolution, indicating an increase in secondary structures.

### SAXS anaxlysis

SAXS is commonly used for the characterization of the low-resolution structure of macromolecules in solution [67, 68]. Importantly, SAXS is especially useful for the analysis of the IDPs with elongated and flexible chains, where other methods fail [69]. Therefore, we performed SAXS studies to get additional information regarding the structure and conformational dynamics of GCEC in solution.

Unfortunately, during irradiation, GCEC exhibited radiation damage, which resulted in protein aggregation. Since the SAXS scattering signal is a function of molecular weight, this technique is sensitive to the presence of even a very small fraction of aggregates (higher oligomers or larger impurities). These phenomena significantly affect the measurement results and make them not interpretable [70]. However, the radiation effect was significantly reduced when we performed the SAXS experiment immediately after the protein purification without any further concentration. To get an insight into the structure of GCEC, we collected three 10 min scans and combined them for further analyzes. Although the GCEC was measured in relatively low concentrations, the collected data were of a good enough quality for low resolution modelling (EOM) and to get an insight into the protein’s structural properties.

**Fig. 4:**
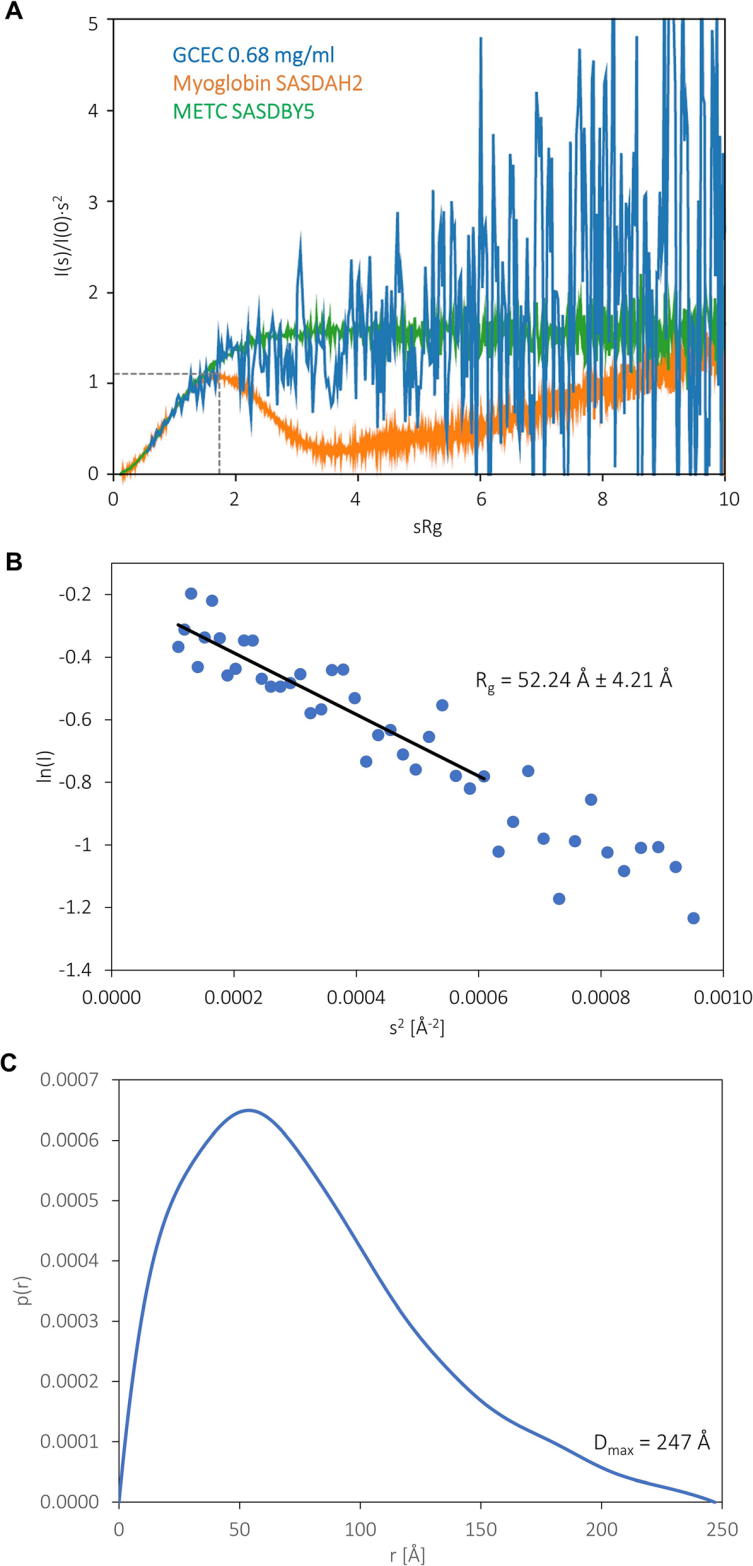
SAXS characteristics of GCEC. **a** Kratky plot analysis [71]. The intensity of scattering is plotted as I(s)/I(0)·s^2^ versus radius of scattering s. The curve representing GCEC SAXS data (blue line) does not have a maximum, and at higher s values reaches a plateau. Two additional curves are presented: for METC (IDP, green line) and for myoglobin (globular protein, orange line). **b** Gunier plot of GCEC SAXS data [71]. The experimental data (blue dots) shows a good fit to the calculated Gunier equation. It indicates monodispersity of GCEC in solution. **c** The pair distribution function p(r) of GCEC. The function is asymmetric, which indicates an elongated GCEC shape

The Kratky plot presents the scattering intensity (I(s)) multiplied by the square of scattering vectors s (s^2^) as a function of the scattering vector s, and was used for SAXS data qualitative analysis [71]. The shape of the Kratky plot is sensitive to protein conformation and is used for the assessment of the protein’s flexibility and degree of its unfolding. The SAXS profile obtained for GCEC (Fig. 4a) does not present the maximum characteristic for globular proteins and reaches a plateau at higher values of the scattering vector. Such a shape is characteristic for IDPs [72]. The Gunier function designated for the GCEC shows a linear character, which is a good indicator of the GCEC’s monodispersity in solution (Fig. 4b). The radius of gyration R_g_ calculated for the GCEC from the Gunier plot (function) was 52.2 Å.

The pair distance distribution function p(r), representing the distribution of all interatomic distances within the molecule [71], was calculated for GCEC using GNOM [46, 73] and experimental SAXS data in the s-range from 0.0104 to 0.1522 Å^− 1^ (Fig. 4c). The R_g_ calculated independently from the Gunier function was 54.1 Å. The maximal intramolecular distance (D_max_) was 247 Å. All the determined parameters indicate the highly asymmetric and expanded GCEC conformation [74].

**Fig. 5:**
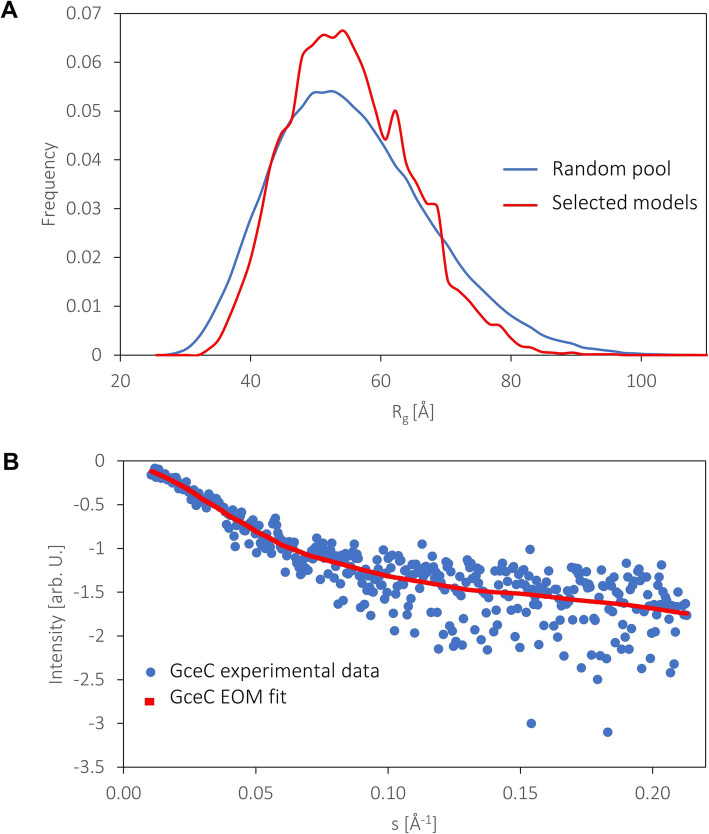
GCEC low resolution structure modeling (EOM). **a** The radii of the gyration profile of the initial random pool of GCEC structures (blue) and the profile of the selected models (red). **b** The fit between the experimental SAXS data (blue dots) and the profile back-calculated from the selected EOM sub-ensemble (red) indicates a good match (χ2 of 0.759)

**Fig. 6:**
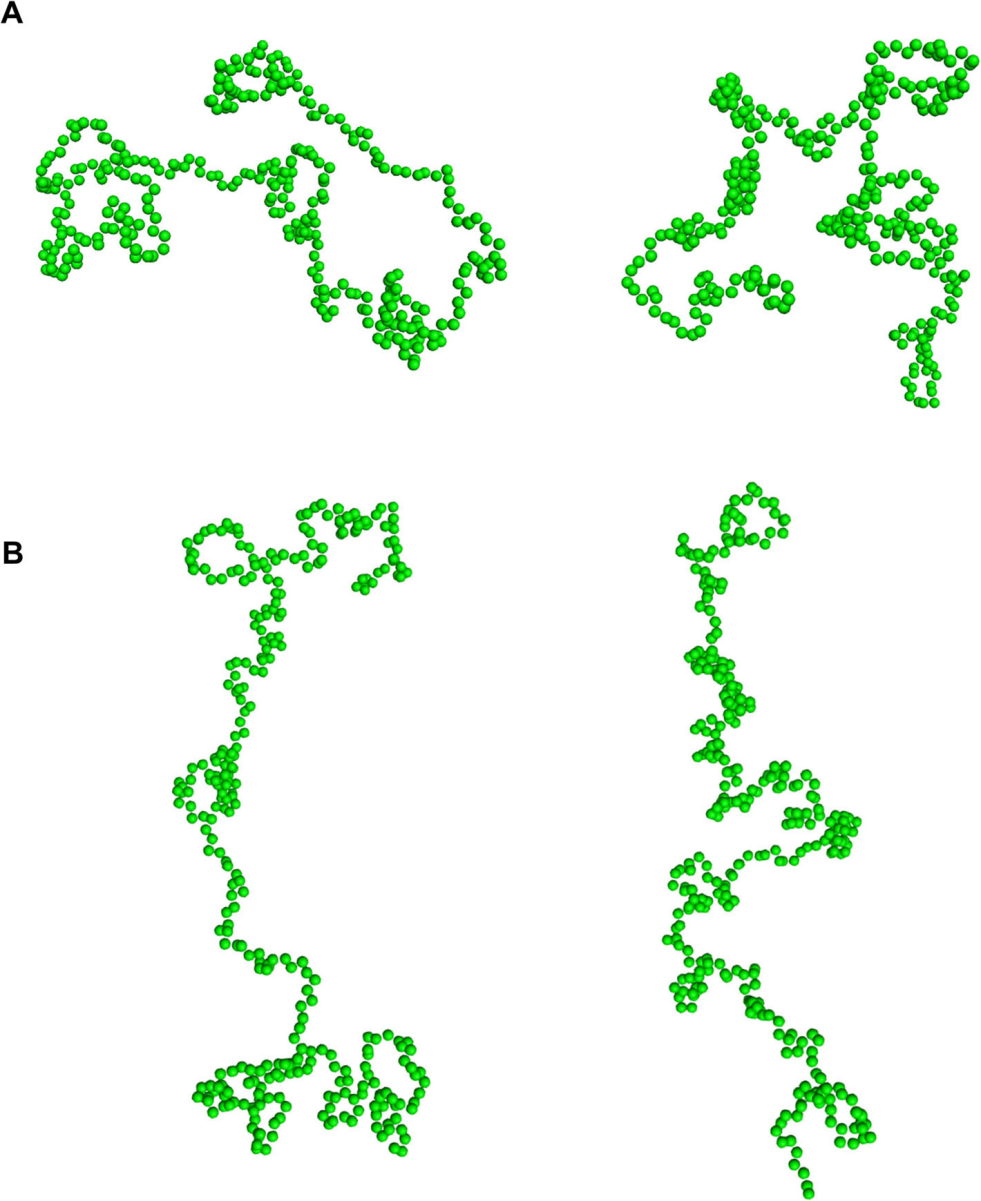
GCEC models. Population of GCEC low resolution models generated with EOM [47]. Two types of models can be distinguished: strongly bent in the middle of the length, and longer, highly tangled at both termini (**b**)

Finally, we performed EOM analysis [47] in order to define the most representative conformations adopted by GCEC in the solution. The EOM algorithm was used to generate a pool of 10,000 random conformers of random coil conformation. Then, a sub-ensemble that fits best to the experimental scattering profile was selected. The R_g_ determined for the final conformational sub-ensemble is slightly moved towards higher R_g_ values (56.0 Å, Fig. 5a) in comparison to the random pool. Moreover, the shape of the histogram is asymmetric and irregular. It means that the sub-ensemble conformations differ in the degree of compaction. An additional peak near 65 Å corresponds to more extended conformations (Fig. 5a). The fit between the experimental data and the back-calculated EOM sub-ensemble is good (χ2 of 0.759) (Fig. 5b). The obtained representative models present two types of conformations: strongly bent in the middle of the length (Fig. 6a), corresponding to the main peak in Fig. 5a, and a longer, highly tangled conformation at both termini (Fig. 6b), corresponding to the additional peak near 65 Å.

### NMR analysis

The interactions comprising full length FTZ-F1 and GCE were studied previously [24]. In our study we focused on the binding capacity between selected protein domains: LBD FTZ-F1, comprising the AF2 motif, and the GCEC region comprising the novel NR-box. Additionally, we tested the interactions between the LBD FTZ-F1 and short GCE^PEP^ peptide (LRLIQNLQK), representing the novel NR-box (LIXXL). We aimed to determine in vitro if the LIXXL motive alone is able to create an interaction surface with the AF2 motif presented in the FTZ-F1.

**Fig. 7:**
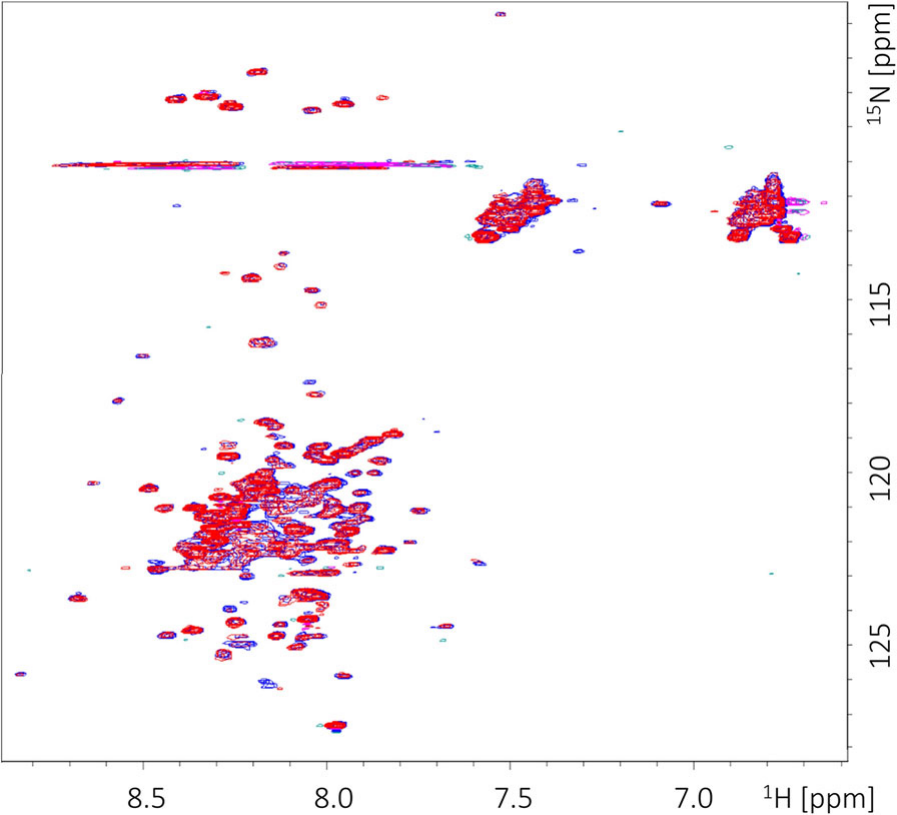
NMR chemical shift perturbation of GCEC by LBD FTZ-F1. ^15^ N HSQC spectra of 100 mM GCEC in the absence (blue) and presence (red) of 100 mM LBD FTZ-F1. The observed chemical shift perturbation indicates the interaction between both proteins

The NMR spectrum of the GCEC was recorded in order to directly confirm the intrinsically disordered character of this protein. The obtained GCEC spectrum is typical for IDPs (Fig. 7; blue). Most proton signals are observed in a narrow frequency range (8–8.5 ppm) and strongly overlap (Fig. 7; blue) with little dispersion. Such a result is caused by the narrow diversity range of the chemical environments experienced by the observed nuclei [75, 76]. The signals of around 6.7 and 7.5 ppm correspond to the side chains of Q and N (Fig. 7; blue). The similar size of all the observed signals confirms the lack of stable, ordered longer fragments in the GCEC structure. The single and more dispersed signals may correspond to the amino acid residues involved in the formation of the short, local and transient motifs of the secondary structure (Fig. 7). The result of NMR analysis is compatible with the above presented CD denaturation data and SAXS experiments. The GCEC spectrum was used as a reference for the chemical shift perturbation experiment, aimed at verifying the GCEC and LBD FTZ-F1 interactions. The adequate spectrum was recorded after incubation with an equimolar amount of unlabeled LBD FTZ-F1. The observed signals show significant changes in intensity (Fig. 7; red). Importantly, multiple signals are shifted, clearly indicating the interaction between proteins (Fig. 7; compare blue and red spectra).

**Fig. 8:**
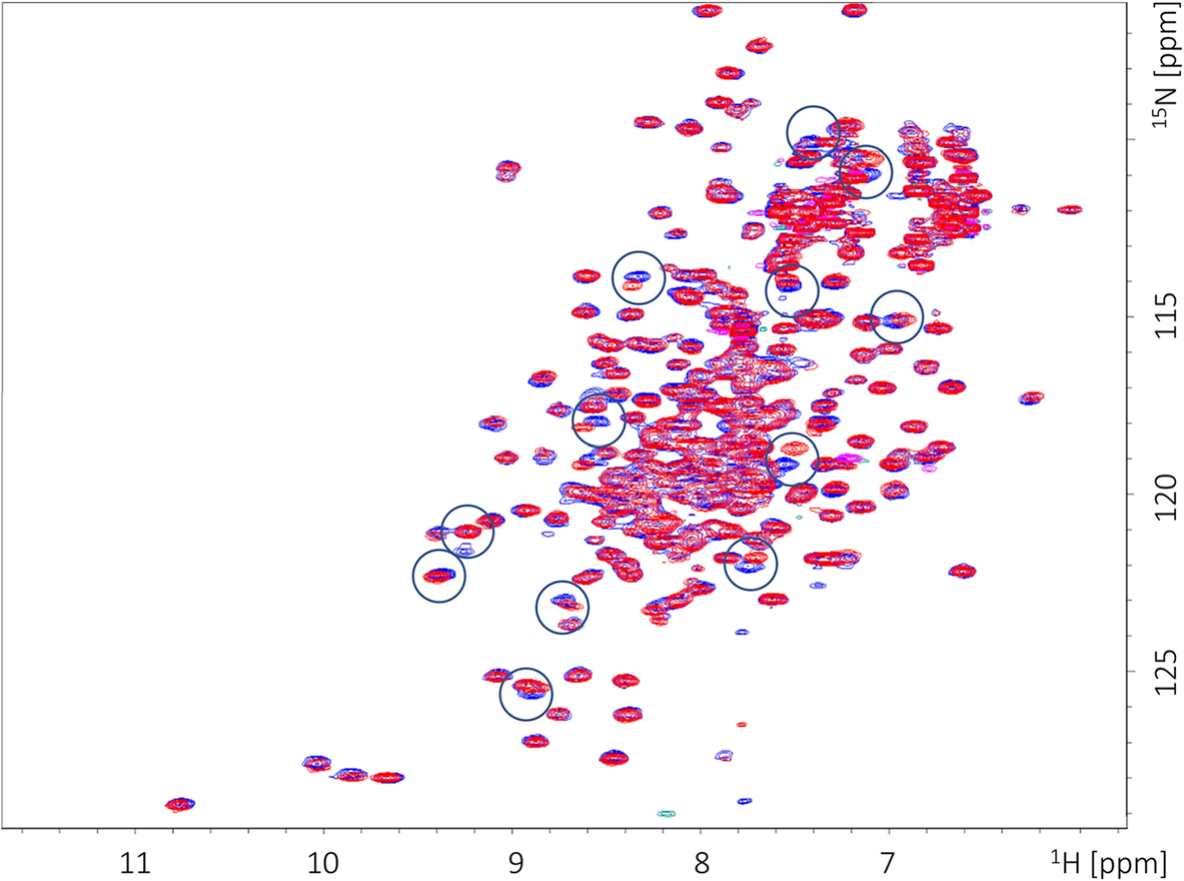
NMR chemical shift perturbation of LBD FTZ-F1 by GCEC^PEP^. ^15^ N HSQC spectra of 100 mM LBD FTZ-F1 in the absence (blue) and presence (red) of GCEC^PEP^. The observed chemical shift perturbation indicates the interaction of LBD FTZ-F1 with a selected fragment (NR box) of GCEC. A selection of the most perturbed cross peaks is marked

We performed an analogical experiment for the labeled LBD FTZ-F1 (Fig. 8, blue). We decided to analyze possible interactions of this domain with short peptide GCEC^PEP^ (LRLIQNLQK), corresponding to the LIXXL motif present in the GCEC sequence [24]. The recorded reference spectrum is similar to the spectrum presented by Daffern et al. [25], and shows a good peaks dispersion appropriate for globular protein (Fig. 8, blue). The spectrum recorded after incubation witch GCE^PEP^ (Fig. 8, red) presents specific signal perturbations and confirms its binding to the LBD FTZ-F1 (Fig. 8; compare blue and red spectra). The specific signals, representing aa residues experiencing major changes, are marked. All the results indicated that the intrinsically disordered GCEC (or GCE^PEP^ representing the novel GCE NR-box) is sufficient to form an interaction interface with the LBD of FTZ-F1 in vitro in the absence of JH. Significantly, these interactions can force GCEC to adopt a more fixed structure. We suggest that GCEC could be sufficient to modulate the FTZ-F1 nuclear receptor activity in the FTZ-F1 LBD dependent manner.

### FLAG pull-down and assay fluorescence analysis

**Fig. 9:**
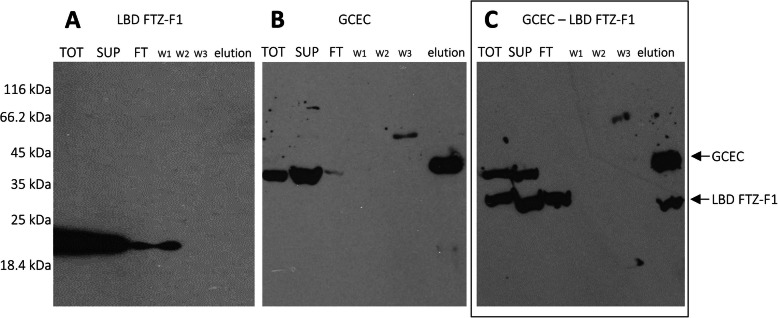
GCEC and LBD FTZ-F1 interact when co-expressed in COS-7 cells. COS-7 cells were transfected with appropriate vectors and pulled-down with ANTI-FLAG M2 Affinity gel. Finally, the samples were analyzed for the presence of GCEC and LBD FTZ-F1 by Western blotting and detected by anti-GFP antibodies. Bands corresponding to YFP-GCEC-FLAG and CFP-LBD FTZ-F1 are marked with arrows. **a** Transfection with the pECFP-C1/LBD FTZ-F1 expression vector. LBD FTZ-F1 is present in cells lysate (TOT), supernatant (SUP) and in the fraction not bound to gel. No protein was observed in the elution fraction. LBD FTZ-F1 does not bind to the ANTI-FLAG M2 Affinity gel. **b** Transfection with the pEYFP-C1/GCEC-FLAG expression vector. GCEC is present in cells lysate (TOT), supernatant (SUP) and the elution fraction. GCEC tagged with FLAG binds to the ANTI-FLAG M2 Affinity gel. **c** Simultaneous transfection with the pECFP-C1/LBD FTZ-F1 and pEYFP-C1/GCEC-FLAG expression vectors. Both proteins (GCEC and FTZ-F1) are detected in the elution fraction. As LBD FTZ-F1 does not possess the FLAG-tag, direct binding to the ANTI-FLAG M2 Affinity gel is not possible and requires interaction with the GCEC-FLAG

To confirm the results of the NMR studies in more natural milieu, i.e. in cells, and to determine the effect of GCEC and LBD FTZ-F1 interactions on the subcellular localization of these proteins, we performed dedicated experiments in COS-7 cells. First, the immunoprecipitation experiment with the ANTI-FLAG M2 Affinity gel was executed. The expressed C-terminally FLAG-tagged GCEC protein or FLAG-tagged GCEC with a partner of interaction (LBD FTZ-F1) were pulled-down from the cell extracts using ANTI-FLAG M2 Affinity gel and then analyzed by Western-blot. The GCEC was additionally tagged on the N-terminus with YFP and LBD-FTZ-F1 with CFP, allowing further localization analyses. Cells transfected with the LBD FTZ-F1 were used as a negative control. The LBD FTZ-F1 with no FLAG-tag is not able to bind to the ANTI-FLAG M2 Affinity gel (Fig. 9a, no bands in the elution fraction). Cells transfected with GCEC-FLAG were used as a positive control. GCEC-FLAG was bound to the ANTI-FLAG M2 Affinity gel and then observed in the immunoblotting as a single band at the appropriate high (Fig. 9b). The observed additional bands are poorly marked and non-specific. When the COS-7 cells were co-transfected with GCEC-FLAG and LBD FTZ-F1, both proteins were observed in the fraction eluted from the ANTI-FLAG M2 Affinity gel and detected with anti-GFP antibody (Fig. 9c). Such a result clearly shows that the intrinsically disordered GCEC comprising the novel NR-box (LIQNL) is sufficient to form an interaction interface with the FTZ-F1 ligand binding domain (LBD) which forms AF2.

Simultaneously, we performed localization studies using proteins of interest N-terminally tagged with YFP (GCEC-FLAG) or CFP (LBD FTZ-F1). As demonstrated by Chalfie et al. [77], green fluorescent protein (GFP) can be used to monitor protein expression and localization in living cells. The labeling of the protein with different fluorescent tags is currently a widely used method that does not affect the localization or the function of fused proteins [78]. Twenty-four hours after transfection (with pEYFP-C1/GCEC-FLAG or pECFP-C1/LBD FTZ F1), or co-transfection (simultaneously with pEYFP-C1/GCEC-FLAG and pECFP-C1/LBD FTZ F1) of the COS-7, we analyzed the expression and subcellular localization of the fluorescently tagged proteins using fluorescent microscopy. While the CFP-LBD FTZ-F1 was distributed within the whole cell (Fig. S5A), YFP-GCEC-FLAG was observed exclusively in the nuclei (Fig. S5A). Simultaneous expression of the GCEC and LBD FTZ-F1 did not affect the GCEC nuclear localization (Fig. S5B), while the LBD FTZ-F1 was shifted to be predominantly nuclear (Fig. S5B). As a control, we transformed COS-7 cells with empty vectors (pEYFP-C1 or pECFP-C1) to express YFP or CFP. As expected, the expression of YFP or CFP resulted in the ubiquitous localization of the proteins (Fig. S5C) and did not influence the fused proteins’ localization.

## Discussion

It was shown that GCE and MET, as JH receptors, mediate hormone action and prevent the precocious development of *D. melanogaster* during metamorphosis [6]. However, their functions are tissue specific and not fully redundant [8]. The most significant difference in the GCE and MET sequences can be observed by the alignment of their C-termini. We hypothesize that it is exactly these regions of both proteins that are responsible for the distinct functions of GCE and MET. This is consistent with the assumption of Furness et al. [79], who stated that the C-termini of bHLH-PAS transcription factors are the key factors in these proteins’ activity regulation. Moreover, Uversky suggested [80] that the long tail is the most important regulatory region for bHLH-PAS proteins.

In this paper we present GCEC as a highly elongated and flexible molecule with residual structural motifs. GCEC can be specified as a coil-like IDP with a high propensity for induced folding. In contrast, METC was defined previously as a PMG-like IDP with a slightly higher structure compactness [27]. C-terminal IDRs are commonly observed in proteins [80] and seem to be a significant aspect of their structural organization, and therefore the protein’s functionality. Importantly, IDPs/IDRs can perform their functions in both a disordered state and after induced folding [80].

During purification, we observed high GCEC aggregation and its significant insolubility in contrast to the previously studied METC [27]. Interestingly, we also observed GCEC oxidation despite using the reducing agent. The oxidation of amino acid residues usually occurs on the M and C residues exposed to the solvent [81]. The covalent addition of oxygen to M alters its hydrophobicity, which may have functional consequences [82]. Importantly, exposed M affect the function and structure of protein since such residues are predisposed to reversible oxidation and reduction reactions [83, 84]. Dynamic changes in these modifications contribute to many important cellular processes or functions in vivo [82]*.* In addition, M and C oxidation can perform antioxidant functions, protecting against the modification of other important amino acid residues critical for protein activity. In the case of human α2 macroglobulin (hα2M), a broad spectrum protease inhibitor, oxidation of exposed M residue prevents modification of the Y residue located in the active center [81, 84]. In the case of GCEC sequencing, analysis indicated two oxidized M: M461 (M71 in GCEC) and M639 (M249 in GCEC). The functional significance of GCEC oxidation and other possible post translational modifications (PTMs) has not been studied and explained to date. However, it is known that the PTMs (like oxidation) can reduce the protein’s half-life time [85]. Moreover, intrinsically disordered chains are degraded much faster than globular proteins [86]. Therefore, oxidation may increase GCE susceptibility to proteolysis, which may explain the cyclical occurrence of GCE in *D. melanogaster* hemolymph.

The presence of short ordered structures in the GCEC sequence was predicted with the PONDR-VLXT server as deep minima in the middle part of long disordered fragments [62, 87], mainly near 30 aa residue, in the area between 150 and 250 aa residues, and near 260 aa residue, considered as MoREs (Fig. 1b). Importantly, the performed CD denaturation experiment and SAXS analysis confirmed the presence of some more ordered fragments in GCEC. The minimum starting near residue 26 of GCEC corresponds to the conserved LIXXL motif enabling interaction with the FTZ-F1 nuclear receptor [24]. What is important is that protein-protein interactions (PPI) usually result in the conformational transitions of MoREs to more ordered forms [62, 87]. The GCEC structure seems to be enriched in short ordered fragments in comparison to METC [27]. Moreover, GCEC exhibits, characteristic for some IDPs, a “turned out” response to heat [63] and adopts a more ordered conformation during the temperature increase. Such behavior was not observed for METC [27]. Accordingly, we suppose that GCEC is an IDR with a much greater propensity for structure ordering, and in consequence it might interact with more physiological partners.

EOM modelling allowed us to select the model conformations adopted by GCEC in the solution. Two types of structures can be distinguished: strongly bent in the middle of the length (Fig. 6a), and longer, highly tangled at both termini (Fig. 6b). GCEC has a hooked structure, which may explain the entanglement and aggregation of its molecules during the purification procedure. The multiplicity of conformations adopted by GCEC is critical for its activity as a biological switch connecting different signaling pathways in insects. Each conformation can determine its specific activity or localization. The ratio between experimentally determined R_g_ and R_S_ is a useful parameter that allows the shape of the protein molecule in solution to be determined [88]. The theoretical value is 0.778 for a hard sphere, from 0.875 to 0.987 for oblate ellipsoids, and from 1.36 to 2.24 for prolate ellipsoids [88]. The value calculated for GCEC was 1.2, while the value calculated for METC was 1.62 [27]. Such a result is consistent with previously published METC characteristics [27] and with the GCEC analysis presented in this paper. METC taking the shape of a highly elongated ellipsoid is characterized by its small diameter in the cross section of the molecule. GCEC, because of the bent in the middle part, reaches a similar length to METC and has a much larger diameter.

The interaction between full length FTZ-F1 and GCE proteins was documented [24]. What is important is that GCE, in contrast to MET, can interact with FTZ-F1 in a hormone independent manner [5]. We asked the question whether the interaction between specific parts of proteins: the FTZ-F1 ligand binding domain (LBD) and GCEC or GCE^PEP^ representing the novel GCE NR-box predicted as MoRE, are also possible. To answer the question we performed NMR spectroscopy studies, which allowed the weak interactions of IDPs and IDRs in vitro [21], supported by the pull-down assay. The presented results were consistent and indicated the interactions between the LBD of FTZ-F1 and GCEC/GCE^PEP^. Importantly, these interactions can force GCEC to adopt a more fixed structure and could modulate the functions of full-length proteins. We suggest that intrinsically disordered GCEC could be sufficient to modulate FTZ-F1 nuclear receptor activity in the FTZ-F1 LBD dependent manner. We hypothesize that GCEC, which is most probably slightly separated from the bHLH and PAS domains, can to some extent act in an independent way. In contrast, METC adheres closely to the core of the protein, and hormone binding is indispensable for its “opening” [27]. Importantly, the conformational changes within the bHLH and PAS domains, induced by ligand binding or interaction, may still trigger structural changes in GCEC, but to a lesser extent than in the case of METC [27].

The presence of NLSs and NESs is responsible for directing protein into the proper subcellular compartment. It was shown previously that the full length FTZ-F1 localizes in the nucleus in different tissues [89]. In this study, we determined the LBD of FTZ-F1 distribution as uniform both in the nucleus and cytoplasm of the cell (Fig. S5A), while GCEC is located exclusively in the nucleus (Fig. S5A), as previously documented [11]. Interestingly, co-expression of the FTZ-F1 LBD with GCEC shifted the FTZ-F1 LBD to prevail slightly in the nucleus (Fig. S5B). Previously, we showed that full length GCE is observed in both compartments of the cell in the absence and the presence of JH. Importantly, mutation in the NLS that is localized in the C-terminal region of the GCE resulted in the protein being present exclusively in the cytoplasm, regardless of the presence or absence of JH [11]. These results clearly indicate that GCEC is an important region that influences the subcellular localization of the entire protein, and thus its function. Any structural changes within the GCEC region can modulate its NLS activity, forcing the translocation of the protein and determining its activity. On the other hand, any changes in GCEC conformation can be propagated over the rest of the protein, affecting its activity. The presence of localization signals, not only within the defined bHLH and PAS domains, but also in the inherently disordered C-terminal region, shows just how complex the system determining the GCE location at a given time is. It emphasizes the importance of protein distribution in the cell [10, 11].

GCE and MET are often referred to as equivalent JH receptors in literature. In our studies, we focused on the identification of the structural differences between these JH receptors. As shown, GCEC and METC differ in the conformation, the degree of compaction, folding propensity and the distribution of NLSs and NESs within the protein. All the mentioned features undoubtedly determine specific functions of the protein. We believe that the transfer of research results directly from one to another protein is a huge misunderstanding. We think that GCE and MET should be considered in future studies as separate research objects with different molecular characteristics and partly different physiological functions.

## Conclusion

In summary, we present the structural characterization of the GCE bHLH-PAS protein C-terminal region (GCEC), referring the obtained data to the first characterized C-terminus of the paralogous protein – MET (METC). The described structural differences between GCEC and METC may be crucial for their distinct functions and expression diversity during the development and maturation of *D. melanogaster*. Both proteins present the highly elongated and asymmetric conformation typical for IDPs. While GCEC is defined as a coil-like IDP, the slightly more compacted METC is characterized as a PMG-like IDP. However, it is GCEC that contains more MoREs and shows a higher propensity to folding. Therefore, GCEC could most probably interact with more physiological partners when compared to METC.

We also determined that the GCEC is sufficient to create an interaction interface with the LBD of the nuclear receptor FTZ-F1. As shown, GCEC comprising the novel NR-box region interacts with LBD FTZ-F1. We hypothesize that GCEC is slightly separated from the bHLH and PAS domains and can act in an independent way to some extent. Thus, GCEC can induce LBD FTZ-F1 transition towards the nucleus and modulate its activity.

The described analysis contributes to a better understanding of the molecular basis of the functions of the C-terminal fragments of the bHLH-PAS family. This is extremely important, since GCE and MET proteins are the first described hormone receptors in this transcription factor family. Our study might be helpful in explaining the relationship between structure (or the lack of structure) and function, as well as the mode of action and the regulation of IDRs.

## Supplementary information

**Additional file 1: Figure S1.** GCEC aa sequence. Amino acid sequence of C-terminal region of GCE (UniProtKB - Q9VXW7) encompassing 661–959 aa area.

**Additional file 2: Figure S2.** GCEC purification. A) Preparative SEC performed on the Superdex 200 10/300 GL column. Fractions selected for SDS-PAGE are indicated by fraction numbers. Fractions containing purified GCEC are marked with the grey color. B) Commassie Brilliant Blue R 250-stained SDS-PAGE analysis of the GCEC samples. Lane 1, molecular mass standards; lane 2, refolded proteins; lane 3, proteins not bound to the Ni^2+^-NTA resin; lanes 4–5, fractions after elution; lane 6, aggregated GCEC protein; lanes 7–8, purified GCEC.

**Additional file 3: Figure S3.** LBD FTZ-F1 purification. A) Preparative SEC performed on the Superdex 75 10/300 GL column. Fractions co’ntaining purified LBD FTZ-F1 protein are marked with the grey color. B) Commassie Brilliant Blue R 250-stained SDS-PAGE analysis of the LBD FTZ-F1 samples. Lane 1, the bacterial protein fraction; lane 2, the soluble protein fraction; lane 3, the fraction of proteins not bound to the Ni^2+^-NTA resin; lane 4, combined elution fractions; lane 5, LBD FTZ-F1 purified with SEC; lane 6, molecular mass standards.

**Additional file 4: Figure S4.** The sequences of primers used in PCR. The primers used for LBD FTZ-F1 and GCEC cDNA amplification introduce restriction site sequences for the selected endonucleases (underlined in the primer sequences). The upper-case letters in the primer sequence represent the sequence present respectively in LBD FTZ-F1 or GCEC. The reverse primer for the GCEC introduced C-terminal FLAG protein sequence (DYKDDDDK, marked in blue).

**Additional file 5: Figure S5.** Subcellular localization of GCEC and FTZ-F1. The subcellular distribution of the YFP-GCEC-FLAG and YFP-LBD FTZ-F1 was analyzed 24 h after the transfection or co-transfection of the COS-7 cells. A) Representative images of the COS-7 cells expressing the YFP-GCEC-FLAG or CFP-LBD FTZ-F1 after single transfection. The YFP-GCEC-FLAG was observed in nuclei, while the CFP-LBD FTZ-F1 localized within the whole cell. B) Representative images of the COS-7 cells expressing the YFP-GCEC-FLAG and CFP-LBD FTZ-F1 after co-transfection. The YFP-GCEC-FLAG was still observed in the nuclei, while the CFP-LBD FTZ-F1 was shifted to a predominantly nuclear localization. C) Representative images of the YFP and CFP expression used as the control. Both proteins were observed within the whole cell.

## Data Availability

All data generated or analysed during this study are included in this published article [and its supplementary information files].

## Abbreviations

20E: 20-hydroxyecdysone
AF2: Activation function 2
AUC: Analytical ultracentrifugation
bHLH-PAS: Basic helix-loop-helix/Per-Arnt-Sim
CD: Circular dichroism spectroscopy
CFP: Cyan fluorescent protein
ECR: Ecdysone receptor
EOM: Ensemble optimization method
FTZ-F1: Nuclear receptor Fushi Tarazu factor-1
GCE: Germ cell-expressed protein
GCEC: C-terminal region of the GCE
GFP: Green fluorescent protein
IDP: Intrinsically disordered protein
JH: Juvenile hormone
LBD: Ligand binding domain
MET: Methoprene tolerant protein
METC: C-terminal region of the MET
MM: Molecular mass
MoREs: Molecular recognition elements
NES: Nuclear export signal
NLS: Nuclear localization signal
NMR: Nuclear magnetic resonance spectroscopy
NR-box: Nuclear receptor box
PTM: Post translational modification
SAXS: Small-angle X-ray scattering
SEC: Size-exclusion chromatography
TAD/RPD: Transcription activation/repression domains
USP: Ultraspiracle
YFP: Yellow fluorescent protein
