# Supplementary figures and images for: The intrinsically disordered region of GCE protein adopts a more fixed structure by interacting with the LBD of the nuclear receptor FTZ-F1
Source: Cell Commun Signal. 2020 Nov 5;18:180. doi: 10.1186/s12964-020-00662-2 (PMC7643343; doi:10.1186/s12964-020-00662-2)

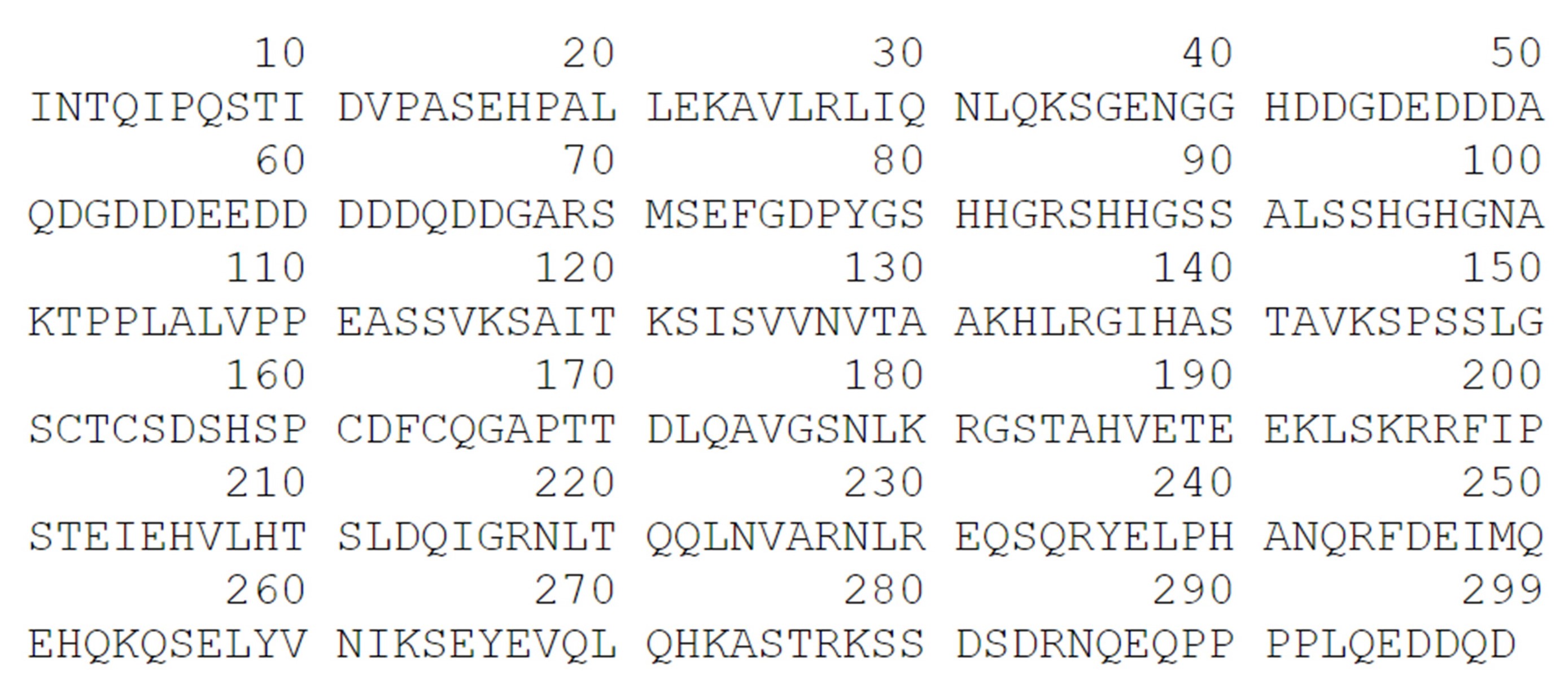

Supplement: Supplementary file 2 — Additional file 1: Figure S1. GCEC aa sequence. Amino acid sequence of C-terminal region of GCE (UniProtKB - Q9VXW7) encompassing 661–959 aa area. [file 12964_2020_662_MOESM1_ESM.jfif]

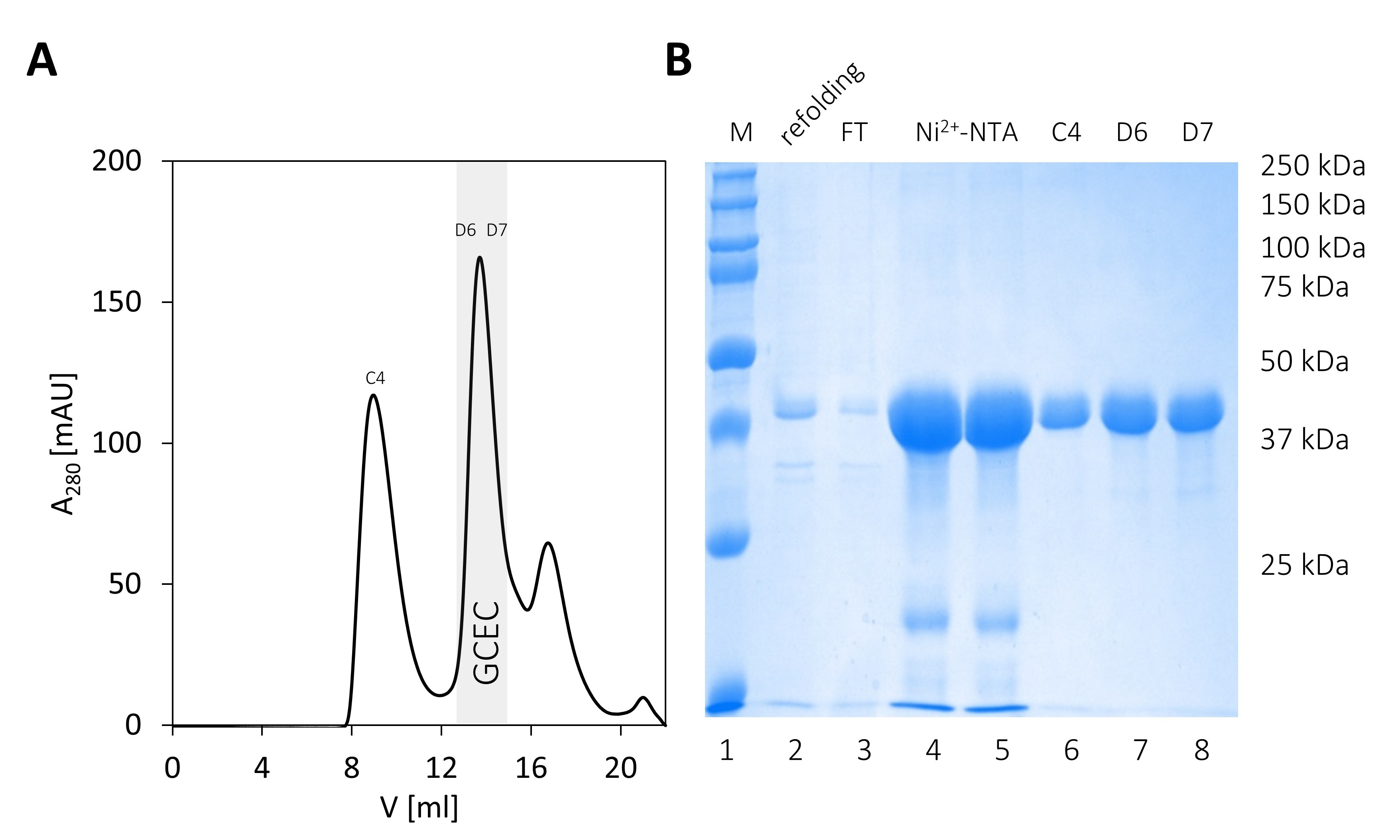

Supplement: Supplementary file 3 — Additional file 2: Figure S2. GCEC purification. A) Preparative SEC performed on the Superdex 200 10/300 GL column. Fractions selected for SDS-PAGE are indicated by fraction numbers. Fractions containing purified GCEC are marked with the grey color. B) Commassie Brilliant Blue R 250-stained SDS-PAGE analysis of the GCEC samples. Lane 1, molecular mass standards; lane 2, refolded proteins; lane 3, proteins not bound to the Ni2+-NTA resin; lanes 4–5, fractions after elution; lane 6, aggregated GCEC protein; lanes 7–8, purified GCEC. [file 12964_2020_662_MOESM2_ESM.jfif]

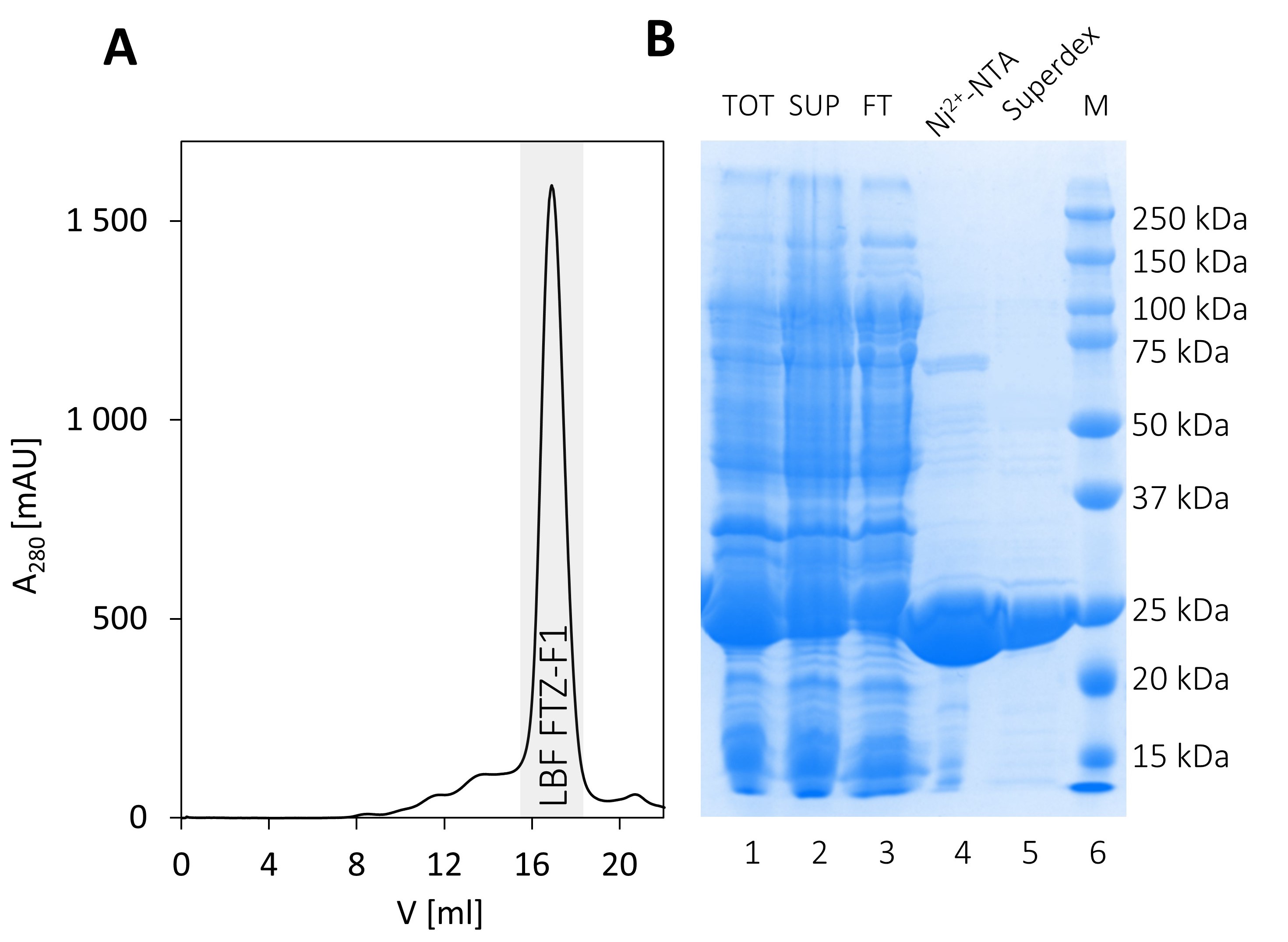

Supplement: Supplementary file 4 — Additional file 3: Figure S3. LBD FTZ-F1 purification. A) Preparative SEC performed on the Superdex 75 10/300 GL column. Fractions co’ntaining purified LBD FTZ-F1 protein are marked with the grey color. B) Commassie Brilliant Blue R 250-stained SDS-PAGE analysis of the LBD FTZ-F1 samples. Lane 1, the bacterial protein fraction; lane 2, the soluble protein fraction; lane 3, the fraction of proteins not bound to the Ni2+-NTA resin; lane 4, combined elution fractions; lane 5, LBD FTZ-F1 purified with SEC; lane 6, molecular mass standards. [file 12964_2020_662_MOESM3_ESM.jfif]

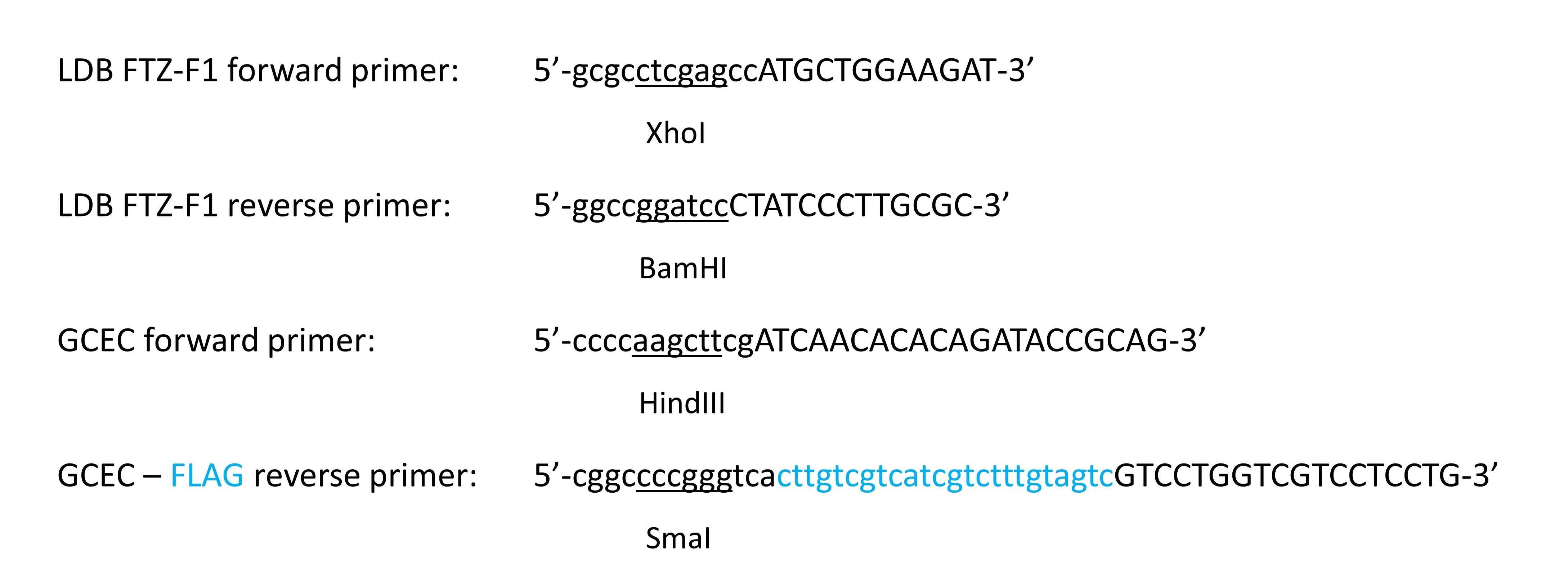

Supplement: Supplementary file 5 — Additional file 4: Figure S4. The sequences of primers used in PCR. The primers used for LBD FTZ-F1 and GCEC cDNA amplification introduce restriction site sequences for the selected endonucleases (underlined in the primer sequences). The upper-case letters in the primer sequence represent the sequence present respectively in LBD FTZ-F1 or GCEC. The reverse primer for the GCEC introduced C-terminal FLAG protein sequence (DYKDDDDK, marked in blue). [file 12964_2020_662_MOESM4_ESM.jfif]

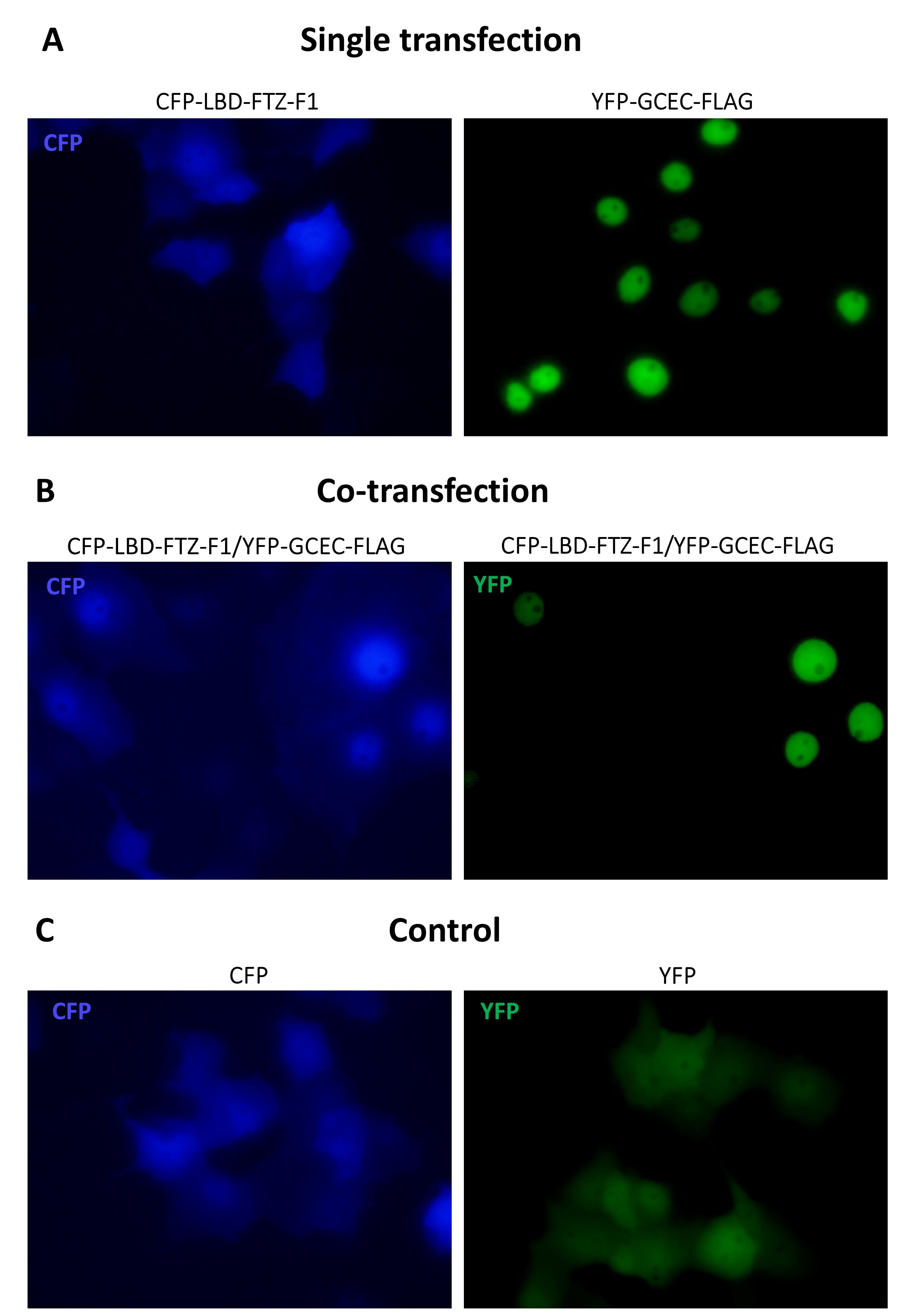

Supplement: Supplementary file 6 — Additional file 5: Figure S5. Subcellular localization of GCEC and FTZ-F1. The subcellular distribution of the YFP-GCEC-FLAG and YFP-LBD FTZ-F1 was analyzed 24 h after the transfection or co-transfection of the COS-7 cells. A) Representative images of the COS-7 cells expressing the YFP-GCEC-FLAG or CFP-LBD FTZ-F1 after single transfection. The YFP-GCEC-FLAG was observed in nuclei, while the CFP-LBD FTZ-F1 localized within the whole cell. B) Representative images of the COS-7 cells expressing the YFP-GCEC-FLAG and CFP-LBD FTZ-F1 after co-transfection. The YFP-GCEC-FLAG was still observed in the nuclei, while the CFP-LBD FTZ-F1 was shifted to a predominantly nuclear localization. C) Representative images of the YFP and CFP expression used as the control. Both proteins were observed within the whole cell. [file 12964_2020_662_MOESM5_ESM.jfif]
